# OSM potentiates preintravasation events, increases CTC counts, and promotes breast cancer metastasis to the lung

**DOI:** 10.1186/s13058-018-0971-5

**Published:** 2018-06-14

**Authors:** Ken Tawara, Celeste Bolin, Jordan Koncinsky, Sujatha Kadaba, Hunter Covert, Caleb Sutherland, Laura Bond, Joseph Kronz, Joel R. Garbow, Cheryl L. Jorcyk

**Affiliations:** 1 Department of Biological Sciences, Biomolecular Sciences Program, Boise State University, 1910 University Drive, Boise, ID 83725 USA; 2 Mercy Medical Center, Nampa, ID USA; 3 Mallinckrodt Institute of Radiology, Washington University, St. Louis, MO 63110 USA

**Keywords:** Oncostatin M, Breast cancer, Metastasis, Circulating tumor cells

## Abstract

**Background:**

Systemic and chronic inflammatory conditions in patients with breast cancer have been associated with reduced patient survival and increased breast cancer aggressiveness. This paper characterizes the role of an inflammatory cytokine, oncostatin M (OSM), in the preintravasation aspects of breast cancer metastasis.

**Methods:**

OSM expression levels in human breast cancer tissue samples were assessed using tissue microarrays, and expression patterns based on clinical stage were assessed. To determine the in vivo role of OSM in breast cancer metastasis to the lung, we used three orthotopic breast cancer mouse models, including a syngeneic 4T1.2 mouse mammary cancer model, the MDA-MB-231 human breast cancer xenograft model, and an OSM-knockout (OSM-KO) mouse model. Progression of metastatic disease was tracked by magnetic resonance imaging and bioluminescence imaging. Endpoint analysis included circulating tumor cell (CTC) counts, lung metastatic burden analysis by qPCR, and ex vivo bioluminescence imaging.

**Results:**

Using tissue microarrays, we found that tumor cell OSM was expressed at the highest levels in ductal carcinoma in situ. This finding suggests that OSM may function during the earlier steps of breast cancer metastasis. In mice bearing MDA-MB-231-Luc2 xenograft tumors, peritumoral injection of recombinant human OSM not only increased metastases to the lung and decreased survival but also increased CTC numbers. To our knowledge, this is the first time that a gp130 family inflammatory cytokine has been shown to directly affect CTC numbers. Using a 4T1.2 syngeneic mouse model of breast cancer, we found that mice bearing 4T1.2-shOSM tumors with knocked down tumor expression of OSM had reduced CTCs, decreased lung metastatic burden, and increased survival compared with mice bearing control tumors. CTC numbers were further reduced in OSM-KO mice bearing the same tumors, demonstrating the importance of both paracrine- and autocrine-produced OSM in this process. In vitro studies further supported the hypothesis that OSM promotes preintravasation aspects of cancer metastasis, because OSM induced both 4T1.2 tumor cell detachment and migration.

**Conclusions:**

Collectively, our findings suggest that OSM plays a crucial role in the early steps of metastatic breast cancer progression, resulting in increased CTCs and lung metastases as well as reduced survival. Therefore, early therapeutic inhibition of OSM in patients with breast cancer may prevent breast cancer metastasis.

**Electronic supplementary material:**

The online version of this article (10.1186/s13058-018-0971-5) contains supplementary material, which is available to authorized users.

## Background

The inflammatory gp130 family of cytokines has been shown to modulate immune function [1] with important implications for tumor immunology [2]. Inflammation and inflammatory cytokines have been associated with increased breast cancer metastasis and poor survival rates [3]. Interleukin-6 (IL-6), a well-known inflammatory cytokine in the gp130 family, promotes breast cancer metastasis [4]. Other cytokines within the gp130 family also modulate inflammation. One such member, oncostatin M (OSM), has been associated with a wide variety of inflammatory disease states, such as in inflammatory bowel disease and arthritis [5, 6]. In the context of cancer, OSM has been shown to induce in vitro effects associated with cancer invasiveness and to promote breast cancer metastasis to bone in vivo [7]. In the breast tumor microenvironment, OSM is produced by breast tumor cells [8], as well as by stromal cells, including tumor-associated macrophages and neutrophils [9, 10]. After the secretion of OSM, OSM binds to and accumulates in the extracellular matrix (ECM) in an active form. This accumulated OSM may then lead to chronic local inflammation and increased tumor metastasis [11]. Specifically, it has been shown that human breast tumor cells signal neutrophils to secrete OSM, which subsequently induces tumor cell vascular endothelial growth factor (VEGF) production, cell detachment, and invasive capacity [9]. Collectively, these studies suggest that OSM functions in breast cancer progression in both an autocrine and a paracrine fashion.

OSM signaling uses two dimeric receptors. OSM binds with high affinity to the OSM receptor (OSMR), which consists of a gp130 subunit and OSMRβ, and with lower affinity to the leukemia inhibitory factor receptor (LIFR), which consists of gp130 and LIFRβ [12]. OSMR signaling initiates the JAK/STAT, mitogen-activated protein kinase (MAPK), and phosphoinositide 3-kinase/AKT pathways [13, 14], as well as the stress-activated MAPK p38 and JNK pathways [15]. Whereas OSM binding to the OSMR has been shown to promote cancer cell malignancy and reduce long-term survival in patients with breast cancer [16], LIF activation of the LIFR suppresses tumor growth and metastasis [17]. Activation of the LIFR by OSM promotes bone growth and may also suppress breast cancer metastatic phenotypes [12, 18].

As a pleiotropic cytokine, OSM appears to play an important role in promoting breast cancer metastatic potential in vitro while inhibiting breast tumor cell proliferation [19]. OSM has been shown to function in breast and various other cancer cells in culture to (1) promote an epithelial-to-mesenchymal transition (EMT) and a stem cell-like phenotype [14, 20]; (2) upregulate expression of proteases such as matrix metalloproteinases [21]; (3) promote tumor cell detachment and subsequent invasion [22, 23]; (4) induce the expression of VEGF, hypoxia inducible factor-1α, and other proangiogenic factors [24]; and (5) suppress estrogen receptor (ER)-α expression [16]. Despite increasing in vitro evidence, limited studies have addressed the role of OSM in breast cancer metastasis in vivo*.*

Our previous studies were the first to show the importance of OSM in breast cancer metastasis to bone. Specifically, reduced tumor cell-produced OSM expression led to a decrease in osteolytic bone metastasis in an orthotopic 4T1.2 mouse model [7]. Because it has been demonstrated that OSM functions in normal bone homeostasis [25], this work suggests an important role for OSM during postintravasation breast cancer metastasis to bone and subsequent bone destruction. Although in vitro studies suggest that OSM promotes the early steps of the metastatic cascade, no existing work differentiates between OSM’s impact on pre- versus postintravasation aspects of the breast cancer metastatic cascade.

The work presented in this paper demonstrates that OSM initiates the preintravasation steps of the metastasis cascade, increases circulating tumor cell (CTC) numbers, promotes lung metastases, and decreases survival in mice. Conversely, we also show that OSM has no effect on survival in the postintravasation model that bypasses the early steps of metastasis by injecting tumor cells directly into the circulation. Collectively, our work suggests that therapeutic suppression of OSM in the tumor microenvironment not only might be an effective treatment strategy for bone metastasis but also could be used as a preventive therapeutic to mitigate overall breast cancer metastasis.

## Methods

### Tissue microarrays

Breast tissue from 72 patients was obtained from paraffin block archives at the Department of Pathology, Mercy Medical Center, Nampa, ID, USA. Three tissue microarrays (TMAs) of 1-mm thickness were assessed. The TMAs were stained for OSM using the Histostain kit (catalogue number 95-9843; Life Technologies, Carlsbad, CA, USA) per the manufacturer’s instructions. The TMAs were deparaffinized and stained overnight with a 1:400 dilution of rabbit antihuman OSM primary antibody (catalogue number sc-129; Santa Cruz Biotechnology, Dallas, TX, USA) and for 1 hour with 1:1000 goat antirabbit IgG-alkaline phosphatase secondary antibody. TMAs stained with secondary antibody alone served as the negative control, and spleen and salivary gland served as positive controls for OSM staining. The TMAs were analyzed in multiple sets of random orders for OSM expression intensity by a pathologist and were graded as follows: 0 = no staining; 1 = light staining; 2 = medium staining; and 3 = dark staining. Grading for each patient was averaged for each cell tissue type (ductal epithelial, vessel, stroma). Additional methods are detailed in Additional file 1: Supplemental Materials and Methods.

### Cell lines and culture conditions

MDA-MB-231-D3H2LN luc2 cells (Caliper Life Sciences, Waltham, MA, USA) and MDA-MB-231 human breast cancer cells (American Type Culture Collection, Manassas, VA, USA) were cultured in RPMI 1640 media supplemented with 10% FBS and 100 U/ml penicillin-streptomycin. Cells were maintained at 37 °C, 5% carbon dioxide, and 95% humidity. 4T1.2 mouse mammary cancer cells [26] were cultured in α-minimal essential medium (α-MEM) supplemented with 10% FBS, 1 mM sodium pyruvate, and 100 U/ml each of penicillin and streptomycin, and the cells were passaged for no more than 6 months. All media and supplements were obtained from HyClone Laboratories (Logan, UT, USA). All cell lines were tested for mycoplasma contamination by routine 4′,6-diamidino-2-phenylindole staining, and experiments were accomplished within ten passages after cell line thawing.

### Plasmid construct design and cell transfection

To transduce MDA-MB-231-Luc2-D3H2LN cells with a tetracycline (TET)-inducible vector, the full-length OSM complementary DNA was cloned into the pLenti6.3/TO/V5-DEST vector (Life Technologies). Lentiviral transduction of the pLenti6.3/TO/V5-DEST+hOSM vector and pLenti3.3/TR vector was performed using the ViraPower™ II Lentiviral Gateway® Expression System (K367-20; Life Technologies) in accordance with the manufacturer’s instructions. Stably transduced cell lines were tested for TET induction of hOSM expression by enzyme-linked immunosorbent assay (ELISA) and Western blot analysis. To create OSM-knockdown 4T1.2 cells, OSM short hairpin RNA (shRNA) and a LacZ shRNA sequences were cloned into the pSilencer 4.1 plasmid (Life Technologies) and stably transfected into 4T1.2 cells as previously described [7]. Two viable OSM-knockdown 4T1.2 cell lines were generated using different shRNA constructs (4T1.2-shOSM1 and 4T1.2-shOSM2) [7].

### ELISA

OSM produced by TET-inducible MDA-MB-231 (MDA^TO/OSM^) cells was tested for in vitro activity. MDA^TO/OSM^ cells were treated with 0.1 mg/ml TET for 48 hours to generate conditioned media (CM) containing OSM. The CM was then applied to parental MDA-MB-231, MDA-MB-231-Luc2, or T47D cells for 30 minutes. Respective cell lysates were then collected from treated cells using PathScan® Sandwich ELISA Lysis Buffer (catalogue number 7018; Cell Signaling Technology, Danvers, MA, USA). The lysates were then run on a PathScan® Phospho-Stat3 (Tyr705) Sandwich ELISA in accordance with the manufacturer’s instructions (catalogue number 7146; Cell Signaling Technology).

To assess OSM concentration in animal serum, whole blood was collected from (MDA^TO/OSM^) tumor xenograft animals at the experimental endpoint and allowed to coagulate for 30 minutes. The coagulated blood was centrifuged at 2500 rpm for 10 minutes, and the upper layer was collected as serum. The serum was then diluted 1:3 in PBS and used in an hOSM ELISA (catalogue number DY295; R&D Systems, Minneapolis MN, USA), which was performed in accordance with the manufacturer’s instructions.

### Western blot analysis

OSM was induced in MDA^TO/OSM^ cells for 48 hours with 0.1 mg/ml TET in 10% FBS RPMI 1640 media. The CM was collected, run on a gel, and blotted onto 0.22-μm polyvinylidene difluoride membranes (EMD Millipore, Billerica, MA, USA). Membranes were blocked using 5% nonfat dry milk diluted in PBS at pH 7.4 with 0.05% Tween 20 (NFDM-PBS-T). Antihuman OSM antibody (catalogue number sc-129; Santa Cruz Biotechnology) was used at 1:1000 dilution in 5% NFDM-PBS-T, and a secondary antirabbit horseradish peroxidase antibody (catalogue number 711-035-152; Jackson ImmunoResearch Laboratories, West Grove, PA, USA) was used at 1:5000 dilution in 5% NFDM- PBS-T.

### Animals and tumor cell injections

All animal experiments were performed in accordance with the local institutional animal care and use committee (IACUCs). Six-week-old female athymic nude mice were used for the xenograft experiments, and 6-week-old female BALB/c mice were used for the syngeneic studies. All mice were obtained from the National Cancer Institute’s Animal Production Facility (Frederick, MD, USA). OSM-knockout (OSM-KO) BALB/c mice were backcrossed from OSM-KO C57BL/6 mice, which were a kind gift from Dr. Peter Donovan, indirectly, through Dr. James Ihle (St. Jude’s Children’s Hospital, Memphis, TN, USA). Animals were backcrossed for at least ten generations, and genotyping was done at each generation to ensure the presence of the knockout allele. Nonsurgical orthotopic injections were performed as described previously with 2.0 × 10^6^ cells diluted in 50 μl of PBS containing 10% medium for the xenograft model and with 1 × 10^5^ cells for the syngeneic models [7]. For all animals, starting at 2 weeks postinjection, tumor length and width were measured with mechanical calipers three times per week, and tumor volume was estimated using the equation tumor volume = (length × width^2^)/2. The survival endpoint was defined by the IACUC as tumor size greater than 20 mm in diameter, 10% or greater weight loss, and/or appearance of cachexia. At the experimental endpoint, animals were killed, and their organs were harvested and examined for any abnormalities. Further analysis specific to each model is described below.

For peritumoral OSM injections, either 50 μl of PBS or 1 μg of recombinant full-length human OSM (PeproTech, Rocky Hill, NJ, USA) diluted in 50 μl of PBS was injected into the area surrounding the tumor three times per week until the endpoint of the experiment. When the tumors became palpable, mice were randomized into groups and began receiving peritumoral injections.

For the TET-OSM-inducible MDA-MB-231 (MDA^TO/OSM^) experiments, the OSM-induced group was given 2% sucrose water containing 0.1 mg/ml TET, whereas the control mice were given just 2% sucrose water until the endpoint of the experiment. To assess blood platelet numbers, blood was collected at the endpoint into ethylenediaminetetraacetic acid (EDTA)-coated tubes (BD Biosciences, San Jose, CA, USA), and a complete blood count was performed by WestVet Veterinary Clinic (Garden City, ID, USA).

### In vivo bioluminescence imaging and tumor progression

Bioluminescence imaging (BLI) of live animals was initiated at 13 days after cell injection and performed weekly. Three to five mice were scanned at one time. Ex vivo organs were also scanned using BLI. Both procedures followed our previously described protocols [27].

### Detection of circulating tumor cells by Alu qPCR

The detection of human CTCs in mouse blood was performed as described previously [28]. A human DNA standard curve was prepared by adding a specified number of human MDA-MB-231 cells into mouse blood, and the DNA was then isolated for use in the qPCR reactions (Additional file 2: Figure S1). Genomic DNA was isolated from 100 μl of whole blood collected from mice at the end of the experiment. DNA was isolated using the DNeasy Blood & Tissue kit (catalogue number 69581; Qiagen, Hilden, Germany) per the manufacturer’s standard instructions. DNA concentrations were normalized between each sample, and 4.5 ng of DNA was added to each 25-μl qPCR reaction. The qPCR reaction mixture was obtained from the SYBR Green GoTaq qPCR Master Mix (catalogue number TM318; Promega, Madison WI, USA), and reaction mixtures were prepared in accordance with the manufacturer’s recommendations. To each reaction, 0.125 μl of 100 μM human Alu and mouse glyceraldehyde 3-phosphate dehydrogenase (*GAPDH*) primers were added. The primer sequences are listed in Additional file 1: Table S1. Reaction conditions were as follows: 50 °C for 2 minutes, 95 °C for 3 minutes, and 40 cycles of (95 °C for 15 minutes, 60 °C for 30 minutes, and 72 °C for 30 minutes. Fluorescence measurements were taken during the annealing temperature stage (60 °C). Cycle threshold (C_t_) values were determined, and the final results were normalized to mouse *GAPDH* signal levels to normalize any sample-to-sample variance in total blood volume and efficiency in total DNA purification.

### Quantitative PCR

For quantitative analysis of lung metastases, lungs dissected from mice bearing mammary tumors were snap-frozen in liquid nitrogen and pulverized into a fine powder. DNA was extracted using an NaCl-Tris-EDTA buffer (100 mM NaCl, 10 mM Tris-HCl, pH 8.0, 1 mM EDTA) containing 20 μg/ml proteinase K and purified by two phenol/chloroform (1:1 vol/vol) extractions followed by ethanol precipitation. The ratio of cancer cells to normal cells was quantified by measuring the neomycin resistance gene (neo^r^) DNA levels versus the vimentin DNA loading control, as described previously [29]. TaqMan PCR was performed on an Applied Biosystems 7500 real-time thermocycler (Thermo Fisher Scientific, Foster City, CA, USA). Probe and primer sequences are listed in Additional file 1: Table S1. The cycling conditions were as follows: 50 °C for 5 minutes, 95 °C for 2 minutes, then 40 cycles of 95 °C for 1 minute and 60 °C for 45 seconds. Fluorescence was measured every cycle after the annealing step, and C_t_ values were calculated. The data were analyzed using the 2^−ΔΔCt^ method [30].

### In vivo magnetic resonance imaging

In vivo MRI experiments were performed using a 4.7-T small-animal magnetic resonance imaging (MRI) scanner equipped with a DirectDrive™ console (Agilent Technologies, Santa Clara, CA, USA). The instrument is built around an Oxford Instruments (Oxford, UK) magnet containing Magnex (Agilent Technologies, Yarnton, UK) actively shielded (21-cm inner diameter, ~ 30 G/cm, ~ 200 ms rise time) gradient coils driven by International Electric Company (Helsinki, Finland) gradient power amplifiers. Respiratory-gated spin-echo MRI studies were collected using a Stark Contrast (Erlangen, Germany) 2.5-cm birdcage radiofrequency coil. Prior to the imaging experiments, mice were anesthetized with isoflurane and were maintained on isoflurane/O_2_ (1–1.5% vol/vol) throughout data collection. The animals’ core body temperature was maintained at 37 ± 1 °C by circulation of warm air through the bore of the magnet. During the imaging experiments, the respiration rates for all mice were regular and ~ 2 s^− 1^. Synchronization of MRI data collection with animal respiration was achieved with a home-built respiratory-gating unit [31], and all images were collected during postexpiratory periods. The imaging parameters were as follows: repetition time = 3 seconds; echo time = 20 milliseconds; field of view = 2.5 cm^2^; data matrix = 128 × 128; slice thickness = 0.5 mm; number of averages = 4. Lung tumors were manually segmented with ImageJ software (rsbweb.nih.gov/ij; National Institutes of Health, Bethesda, MD, USA), and the number and volume of all metastatic tumors were measured and recorded on an animal-by-animal basis as described previously [32, 33].

### Detection of circulating tumor cells (clonogenic assay)

Colony-forming assays were performed to detect CTCs in mouse blood. At the endpoint of the animal experiment, whole blood was collected into EDTA-coated tubes via intracardiac puncture. Red blood cells (RBCs) were lysed with RBC lysis solution (155 mM NH_4_Cl, 10 mM KHCO_3_, 0.1 mM EDTA diluted in double-distilled H_2_O) for 4 minutes. The remaining cell mixture, containing white blood cells and CTCs, was spun down and washed twice with PBS. The cell pellet was then resuspended in α-MEM with 10% FBS, then plated and incubated at 37 °C for 7–10 days until colonies formed. The colonies were then fixed with 10% formalin in PBS for 15 minutes, stained with Coomassie blue, and counted.

### Epithelial-to-mesenchymal transition assay

4T1.2 mouse mammary cancer cells were plated on a 6-well plate to a confluence of 30% in α-MEM with 10% FBS and 1% penicillin-streptomycin. Following a period of 24 hours to allow cells to adhere, 25 ng/ml recombinant mouse OSM (rmOSM) was added to appropriate wells. Photomicrographs were taken at a power of 100× at 24 and 48 hours to observe phenotypic EMT changes over the 2-day period.

### Cell migration assay

4T1.2 cells were plated on 6-well plates to a confluency of 80% in α-MEM with 10% FBS. After the cells attached overnight, a straight scratch on the cell monolayer was made with a sterile 1000-μl polypropylene pipette tip, and loose cells and debris were washed away with three sterile PBS washes. The cells were then treated with or without 25 ng/ml rmOSM and imaged every day for 3 days on the same part of the scratch using negative phase-contrast microscopy. The images were then imported into ImageJ software, and raw unmigrated area was measured by calculating the number of pixels in the area with no migration. Relative migration intensity was calculated as migration intensity = (day 0 unmigrated area/day *n* unmigrated area) − 1.

### Cell detachment assay

4T1.2 mouse mammary cancer cells were plated on 24-well tissue culture dishes to a confluency of 80% in α-MEM with 10% FBS. The cells were allowed to attach overnight, and rmOSM (25 ng/ml; R&D Systems) suspended in α-MEM with 10% FBS was added to the cells. For up to 8 days, cells that were detached were collected and counted using a hemocytometer, and viable cells were detected by lack of trypan blue staining (catalogue number SV30084.01; HyClone Laboratories).

### Statistical analysis

TMA mean staining values were analyzed with repeated measures in a mixed model framework using compound symmetric covariance within patients and cancer status as fixed effects. All other statistical comparisons between multiple groups were assessed by one- or two-way analysis of variance (ANOVA) using Tukey’s posttest analysis. Comparisons between two groups were analyzed by Student’s *t* test (two-tailed, unpaired). The statistical analyses were performed using Prism version 5.0b (GraphPad Software Inc., La Jolla, CA, USA) or SAS version 9.1.3 (SAS Institute, Cary, NC, USA) software. Survival data were analyzed using the log-rank (Mantel-Cox) test. Significance denoted as *p < 0.05, **p < 0.01, and ***p < 0.001.

## Results

### High OSM expression in ductal carcinoma in situ and invasive ductal carcinoma suggests autocrine signaling

**Fig. 1:**
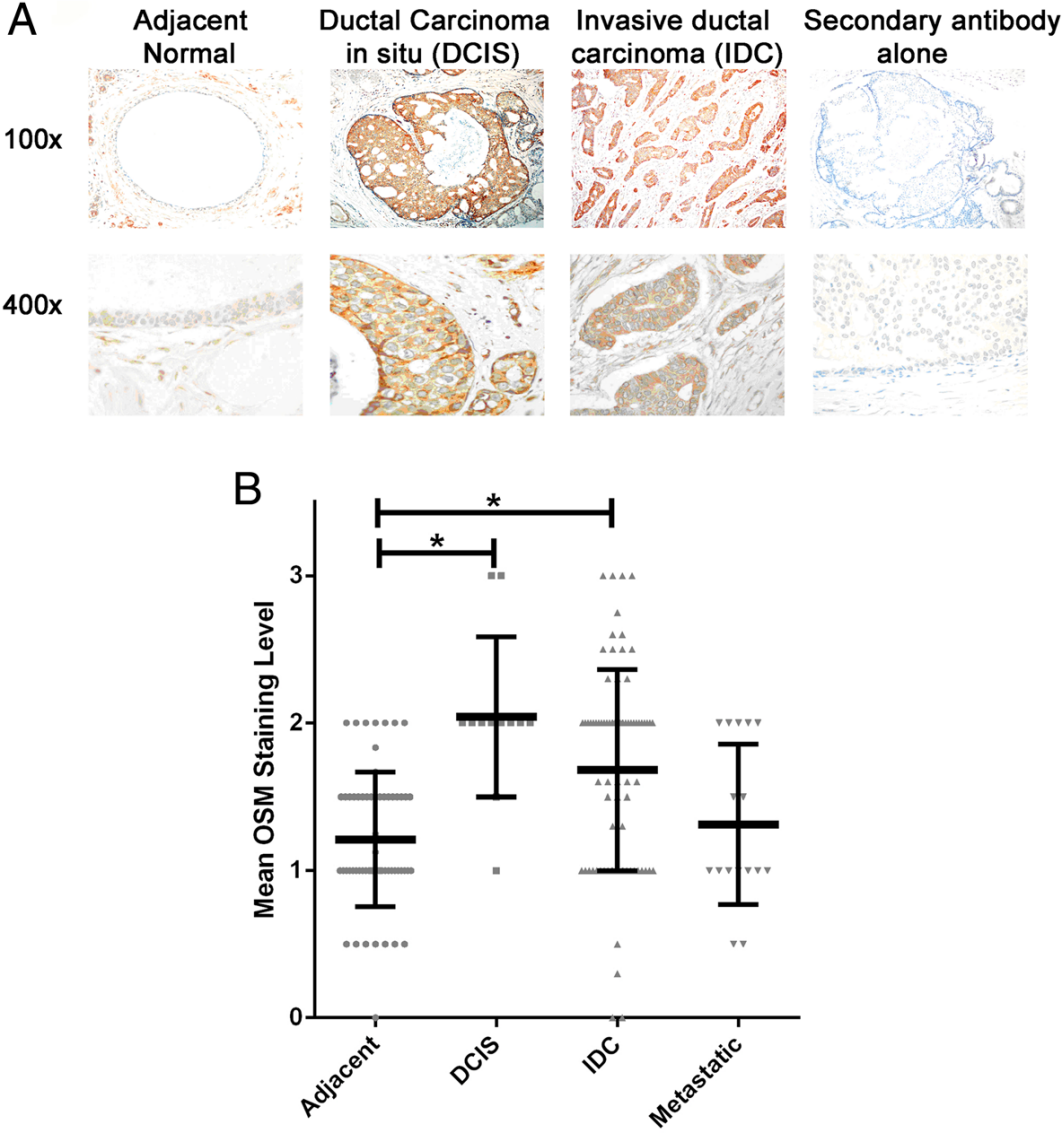
Oncostatin M (OSM) is highly expressed in ductal carcinoma in situ (DCIS) and invasive ductal carcinoma (IDC). **a** To detect the presence of OSM in breast cancer tissue, histological microarrays from 72 patients with breast cancer were stained with human OSM antibody by IHC. Twelve patients had in situ DCIS, 54 patients had nonmetastatic IDC, and 16 patients had IDC with metastasis to lymph nodes (see Additional file 1). The results showed that normal adjacent tissue expresses little OSM but that OSM is highly expressed in DCIS and IDC. Secondary antibody alone did not produce any background signals. **b** Intensity quantification of OSM stained tissues. Mean staining intensity for DCIS (2.00) and IDC (1.66) tissues was significantly higher than that of normal adjacent tissue (1.33) and metastatic tissue (1.24). There was no statistically significant difference between normal and metastatic tissue. Multiple cores from the same patients were averaged. Data are expressed as mean ± SD. *p < 0.05 by one-way analysis of variance with Tukey’s multiple comparisons test

To assess breast epithelial cell expression and location of OSM in human breast tumors, TMAs containing samples from 72 patients were analyzed by IHC. Interestingly, OSM staining intensity was higher in the ductal carcinoma in situ (DCIS) tissue than in normal tissue, whereas no staining was observed using the control secondary antibody (Fig. 1a). Representative images of staining intensity are shown in Additional file 3: Figure S2. Quantification of OSM levels from all sections showed that the mean staining intensity for normal adjacent tissue (1.33) was significantly lower than that of DCIS (2.00) and invasive ductal carcinoma (IDC) (1.66) tissues, whereas metastatic tissue (1.24) was statistically similar to normal tissue (Fig. 1b and Additional file 1: Table S2). Fibroblast OSM expression in the cancerous stroma was significantly lower (0.39 mean OSM staining) than in adjacent normal stroma (0.94 mean OSM staining) (*p* < 0.001) (Additional file 1: Table S3). Additionally, OSM expression was significantly higher in patients with metastasis and those with a positive margin status than in those with a negative margin status (Additional file 1: Table S4). Similarly to stromal OSM expression, we found that endothelial cells of blood vessels near the cancerous tissue had significantly lower OSM expression (1.22 mean OSM staining) than the blood vessel endothelium (1.66 mean OSM staining) around adjacent normal tissue (*p* < 0.001) (Additional file 1: Table S3).

On one hand, breast cancer subtype analysis of IHC staining revealed that OSM expression increased linearly with respect to HER2/Neu status in patients with IDC with increasing HER2/Neu expression (Additional file 1: Table S4). On the other hand, OSM expression levels did not change with respect to HER2/Neu in patients with metastatic disease (Additional file 1: Table S4). Additionally, OSM expression increased slightly (0.2) for every 50% increase in the expression of the ER in patients with IDC but did not change significantly in metastatic tissue. Together, these results suggest that OSM protein levels are higher in the earlier stages of breast cancer and that tumor cell-produced OSM may be important in autocrine signaling for the promotion of tumor progression. Although there is some indication that the expression of OSM is greater in ER+ and HER2+ breast cancer tissue samples, cell lines representing these subtypes have poor tumorigenic and metastatic capacity in vivo [34]. Furthermore, significant OSM expression is present in ER− and HER2/Neu tumors. Therefore, we used metastatic in vivo models based on the MDA-MB-231-Luc2-D3H2LN cell line [35] and the highly metastatic 4T1.2 model, which are both ER−/HER2− [7, 26].

### Elevated production of OSM generated from TET-inducible MDA-MB-231 (MDA^TO/OSM^) cells increases metastases to lungs and decreases overall survival

**Fig. 2:**
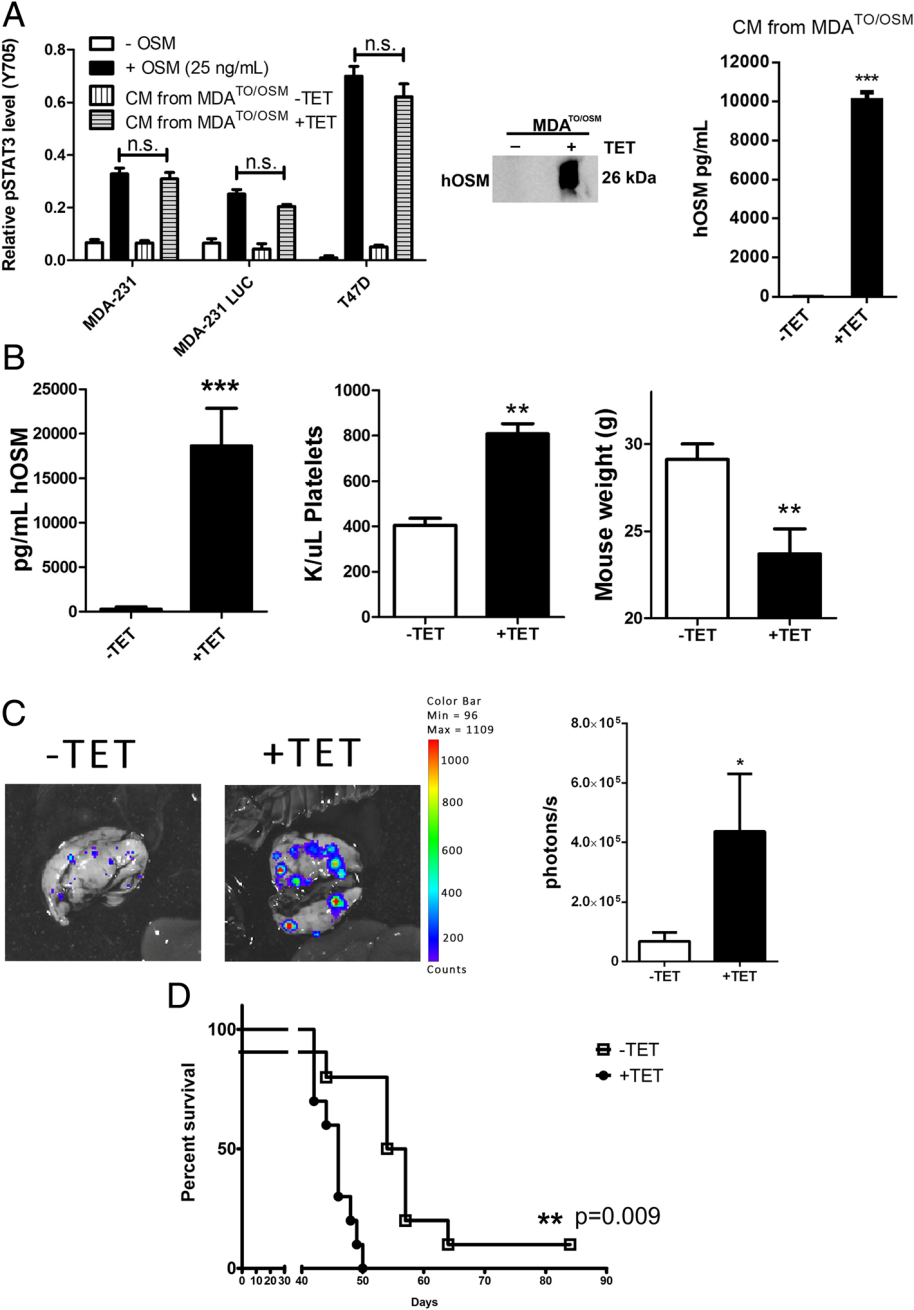
Oncostatin M (OSM) produced by tetracycline (TET)-inducible MDA-MB-231 (MDA^TO/OSM^) cell MDA^TO/OSM^) tumors increase metastasis and decrease survival. **a** MDA^TO/OSM^ human breast cancer cells were treated with (+TET) or without TET (−TET), and the resultant conditioned media (CM) from the treated cells were applied to parental MDA-MB-231, MDA-MB-231-LUC, and T47D cells. *Left*: The activity of OSM accumulated in the CM was compared with commercially obtained recombinant human OSM (rhOSM) (25 ng/ml). There was no significant difference between OSM produced by MDA^TO/OSM^ versus rhOSM versus its ability to induce pSTAT3. *Middle*: Western blot analysis depicting that CM produced by MDA^TO/OSM^ cells stimulated with TET contain OSM. *Right*: Enzyme-linked immunosorbent assay (ELISA) analysis showed that CM from TET-treated MDA^TO/OSM^ cells contain 10.1 ng/ml of hOSM. **b** *Left*: Animals with MDA^TO/OSM^ tumors were given drinking water with or without TET, and whole blood was collected at the experimental endpoint. After allowing the blood to clot and serum was separated by centrifugation, the resultant serum OSM levels were measured by ELISA. Animals with MDA^TO/OSM^ tumors with drinking water containing TET had 67-fold higher serum OSM levels. *Center*: Platelet counts were higher in +TET MDA^TO/OSM^ tumor-bearing mice than in −TET mice. *Right*: +TET MDA^TO/OSM^ tumor-bearing mice had lower body weight than −TET mice. **c** Animals with MDA^TO/OSM^ tumors were given drinking water containing TET for 1 week, and their lung metastasis levels were assessed by ex vivo bioluminescence imaging. *Left*: Representative ex vivo bioluminescence image. *Right*: Average radiance analysis of the ex vivo bioluminescence imaging in photons per second per square centimeter per square radian (p/s/cm^2^/sr). Animals with MDA^TO/OSM^ tumors +TET had a fivefold higher bioluminescent radiance than −TET mice (−TET, *n* = 3; +TET, *n* = 6). Data are expressed as mean ± SEM. **d** Kaplan-Meier survival curve for mice with MDA^TO/OSM^ tumors ± TET. Mice that did not receive TET survived, on average, 11 days longer (−TET, *n* = 9; +TET, *n* = 10). ***p < 0.001 by log-rank test. Data are expressed as mean ± SEM. *p < 0.05, **p < 0.01, and ***p < 0.001 by two-tailed *t* test or one-way analysis of variance with Tukey’s posttest where appropriate

In order to assess the effects of cancer cell-produced OSM in a tumor microenvironment, we developed a stably transduced triple-negative breast cancer (TNBC) MDA-MB-231-Luc2-D3H2LN cell line that secretes OSM in response to TET treatment (+TET;MDA^TO/OSM^). MDA-MB-231-Luc2-D3H2LN cells have enhanced capacity to metastasize to multiple organs, including lung and bone, compared with the parental MDA-MB-231 cells, which have poor metastatic capacity via orthotopic application [35]. To compare the OSM produced by the MDA^TO/OSM^ cells with recombinant human OSM (hOSM), CM from MDA^TO/OSM^ cells treated with TET (0.1 μg/ml) was collected. MDA-MB-231, MDA-MB-231-Luc, and T47D breast cancer cells were treated with either CM from MDA^TO/OSM^ cells or with rhOSM (25 ng/ml) for 30 minutes. OSM signaling was assessed by measuring STAT3 activation using a pSTAT3 ELISA. For each cell line investigated, there were no significant differences in the levels of pSTAT3 induced by OSM produced from TET-induced MDA^TO/OSM^ cells compared with rhOSM (Fig. 2a, left). Furthermore, the CM from MDA^TO/OSM^ cells treated with TET was assessed on an hOSM immunoblot, and an expected size band (26 kDa) was detected (Fig. 2a, middle) by ELISA (Fig. 2a, right), showing an OSM concentration of 10 ng/ml in the CM.

To assess the activity of MDA^TO/OSM^ cells in vivo, 1 × 10^6^ cells were injected into the fourth mammary fat pad of female athymic nude mice. The mice were given drinking water with TET (+TET) or without TET (−TET) (0.1 mg/ml in 2% sucrose water) to induce OSM expression in the cancer cells. At the experimental endpoint, serum was then separated from whole blood, and serum OSM levels were assessed by ELISA. Tumor-bearing mice +TET had a 67-fold higher level of OSM present in their serum than −TET mice (Fig. 2b, left). Each animal’s physical condition was assessed, and animals with MDA^TO/OSM^ tumors +TET had significantly increased blood platelet counts (Fig. 2b, center). TET-treated mice also had a significant decrease in body weight compared with −TET mice (Fig. 2b, right; Additional file 4: Figure S3A), displaying a prominent spinal column and reduced apparent body fat, indicative of cachexia (Additional file 4: Figure S3B). It was previously reported that cachexia, elevated inflammatory factors, and kidney disease may be correlated with each other [36]. In this study, the cachexic animals had kidney abnormalities with hypoperfusion and damage to the gross morphological kidney structures (Additional file 4: Figure S3C). This correlated with +TET treatment and high levels of serum OSM in the animals (Additional file 4: Figure S3D and S3E), suggesting that high OSM levels may contribute to the development of cachexia and kidney dysfunction. Control animals without tumors treated with TET had normal body condition and normal kidney morphology (data not shown).

Additionally, in a separate experiment, animals were given TET drinking water for only 1 week so that we could assess the early effects of OSM on metastasis. Animals receiving 1 week +TET had higher levels of metastases to the lung than −TET mice, as assessed by ex vivo imaging, (Fig. 2c). Only sporadic spine or liver metastases were detected at this early time point (data not shown), which suggests that bone and liver metastases may grow more slowly or occur as a later event. This result also suggests that a short-term elevation in the level of OSM can promote the development of metastases to the lung. To measure animal survival, mice with MDA^TO/OSM^ tumors were treated with or without TET and allowed to progress to the endpoint. Mice given +TET drinking water had a mean decreased survival of 11 days compared with −TET mice (Fig. 2d). Collectively, these results demonstrated that elevated levels of tumor cell-produced OSM led to increased lung metastases, decreased survival, and deterioration in body condition indicative of cachexia.

### OSM increases lung metastases and circulating tumor cell numbers in an orthotopic MDA-MB-231 model of breast cancer

**Fig. 3:**
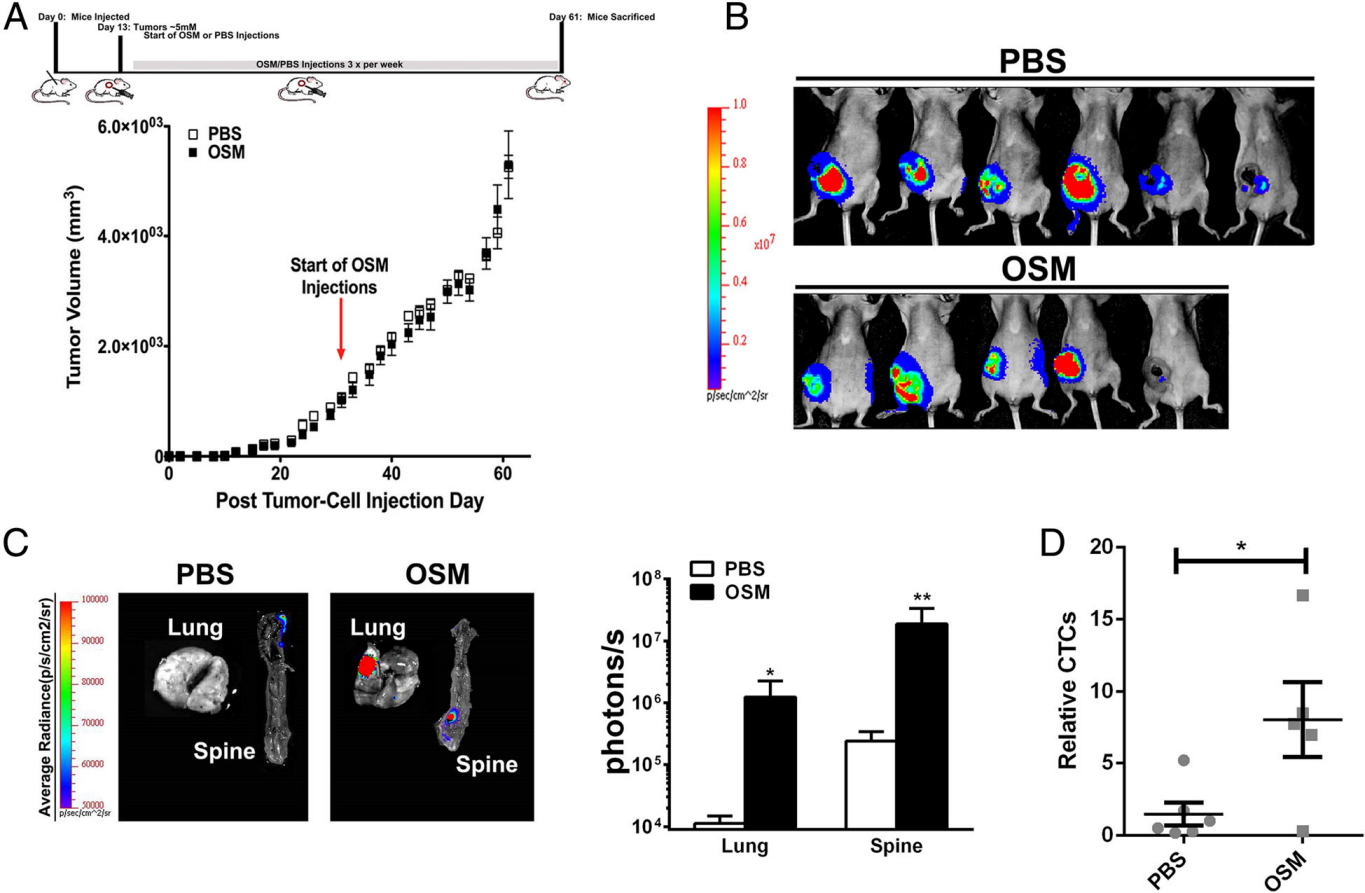
Peritumoral oncostatin M (OSM) injections into mice with MDA-MB-231-D3H2LN tumors promote the development of metastases and circulating tumor cells (CTCs). **a** The timeline shows orthotopic MDA-MB-231-D3H2LN human breast tumor cell injection at day 0, peritumoral OSM or PBS injections beginning three times per week at day 13, and the final day of killing of both groups at day 61. Average tumor volume (mm^3^) did not differ between the peritumorally injected OSM and PBS control groups. **b** Representative images of PBS- and OSM-injected tumor-bearing mice imaged ventrally by bioluminescence imaging (BLI). There was no statistically significant difference between the groups. **c** *Left*: Representative ex vivo BLI study of lungs and spine from mice bearing MDA-MB-231-D3H2LN Luc2 tumors that were treated with peritumoral injections of PBS or OSM. *Right*: Ex vivo BLI intensities were quantified in the lung and spine. Lungs from mice receiving peritumoral OSM injections showed a 37.9-fold higher BLI intensity than those with PBS injections, and spines from mice injected with OSM showed an approximately 25.9-fold increase over mice that received PBS injections. Data are expressed as photons/second (mean ± SEM; *n* = 5–6). **d** Human CTCs containing human Alu DNA were detected in mouse blood by qPCR. In animals that received OSM injections, there was a fourfold increase in the number of CTCs compared with controls. Data are expressed as mean ± SEM *p < 0.05 by two-tailed t test

To assess the paracrine effects of OSM, exogenous OSM was injected peritumorally in an orthotopic MDA-MB-231 xenograft model. In this model, 2 × 10^6^ MDA-MB-231-Luc2-D3H2LN cells were injected into the fourth mammary fat pads of female nude mice. After the tumors were palpable (~ 3 mm), OSM (1 μg in 50 μl of PBS) or PBS alone was injected peritumorally three times per week, and mice were monitored until the endpoint criteria were met. Peritumoral injections can potentially cause supraphysiologic concentrations of OSM in the local tumor microenvironment, which may amplify any effect that OSM has on the tumor. However, in light of recent data suggesting that OSM accumulates in the acidic ECM, actual concentrations of inflammatory cytokines in the tumor microenvironment may be much higher locally than previously thought [11, 37]. Unexpectedly, tumor volume did not differ between the groups (Fig. 3a), even though OSM has been shown to reduce MDA-MB-231 cell proliferation in vitro [38]. The BLI intensities of the tumors from both groups were similar (Fig. 3b), although a few mice in each group had lower BLI intensities owing to tumor necrosis.

Mice receiving peritumoral OSM showed larger metastatic volumes in both lung and spine than mice receiving PBS, as assessed by ex vivo imaging (Fig. 3c, left). Additionally, lungs dissected from the OSM-injected group had BLI intensities that were two orders of magnitude (10^2^) higher than those in the PBS-injected group (Fig. 3c, right). Similarly, spinal BLI intensity from OSM-treated mice averaged 2 × 10^7^ photons/second, whereas control mice had a mean signal of 3 × 10^5^ photons/second.

In patients with advanced and/or inflammatory breast cancer, high numbers of CTCs have been detected, suggesting a correlation between inflammatory factors and the number of CTCs [39]. In our xenograft model, both human MDA-MB-231-Luc2 tumor cells and potential CTCs contained multiple copies of human Alu DNA repeat sequences. To assess CTC numbers, DNA was isolated from mouse blood, and the levels of human Alu DNA repeat sequences were determined in the blood by qPCR. The resultant C_t_ values were fitted to the standard curve to determine total CTC numbers in each sample (Additional file 2: Figure S1). In animals that received rhOSM injections, there was a fourfold increase in the number of CTCs per 100 μl of mouse blood compared with animals that did not receive OSM (Fig. 3d). Collectively, this suggests that increased paracrine OSM in the tumor microenvironment increases metastasis to lung and spine while also increasing CTCs.

### Suppression of OSM in a syngeneic mouse model reduced lung metastases

To use an immunocompetent mouse model, we employed two highly metastatic 4T1.2 mouse mammary tumor cell lines exhibiting knockdown expression of OSM in a syngeneic, orthotopic model of breast cancer [7]. These two independent cell lines (4T1.2-shOSM1 and 4T1.2-shOSM2), developed from two independent shRNA constructs, were shown by ELISA to secrete a 3- to 12-fold reduction in OSM, respectively, compared with control 4T1.2-LacZ cells [7]. To test the effects of OSM on mammary tumor metastasis in vivo, 1 × 10^5^ control 4T1.2-LacZ, 4T1.2-shOSM1, and 4T1.2-shOSM2 cells were injected orthotopically into the mammary fat pads of female BALB/c mice.

**Fig. 4:**
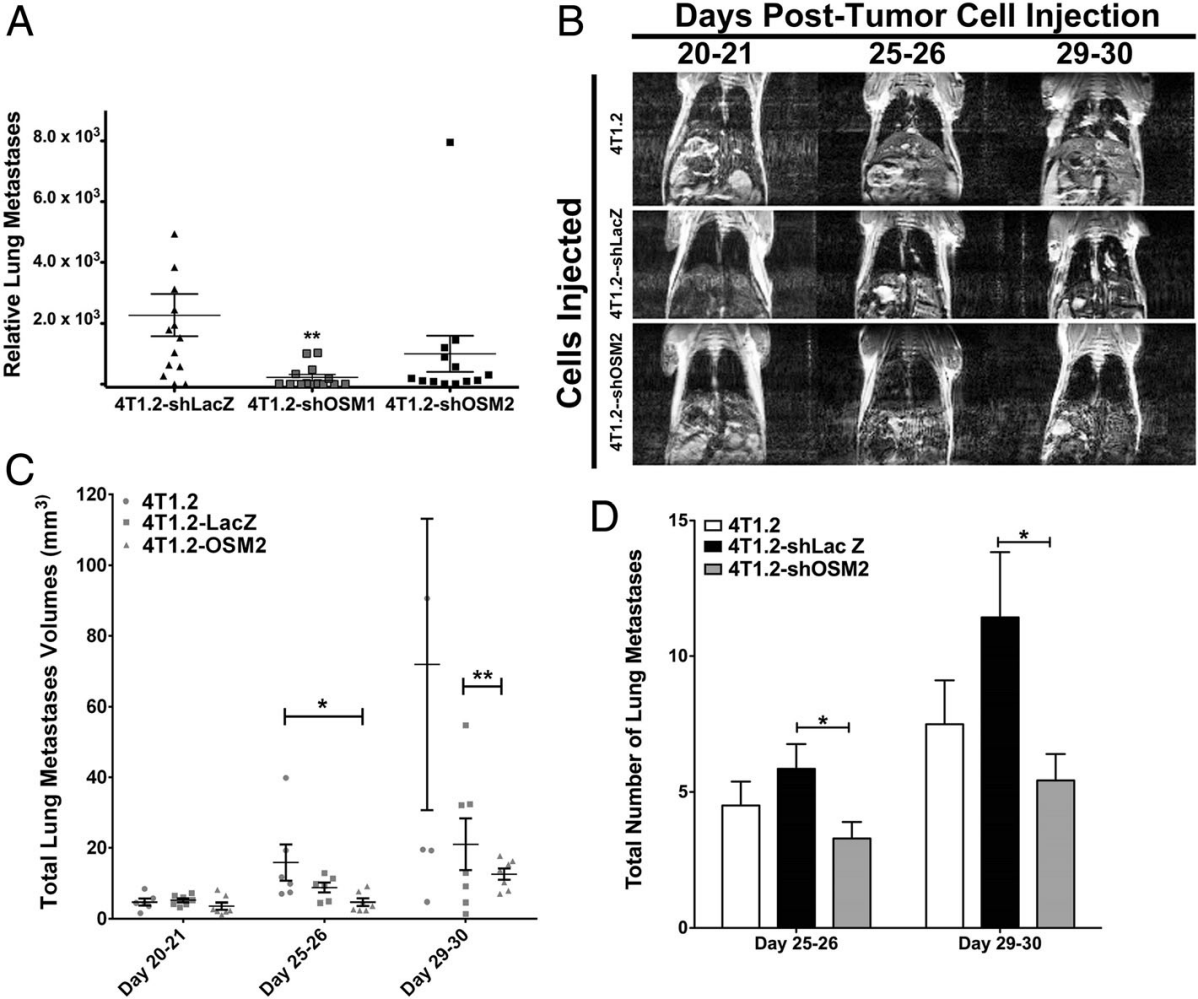
Reduced oncostatin M (OSM) expression results in fewer spontaneous lung metastases and lower total volume of lung metastases by magnetic resonance imaging (MRI). **a** Lung metastasis burden was quantified by qPCR. Mice bearing mammary 4T1.2-shOSM1 or 4T1.2-shOSM2 tumors had less metastasis to the lung than mice with 4T1.2-shLacZ tumors. **b** Mice bearing 4T1.2-shOSM2 tumors had less metastatic lesions in the lung as detected by MRI at the endpoint of the experiment than mice bearing parental 4T1.2 or control 4T1.2-shLacZ tumors. MRI quantification of lung metastatic volume (**c**) and total number of lung metastases (**d**) showed significantly higher volume and number of lung metastases in the 4T1.2- or 4T1.2-shLacZ-injected mice than the 4T1.2-shOSM2-injected mice (4T1.2, *n* = 6; 4T1.2-shLacZ, *n* = 7; 4T1.2-shOSM2, *n* = 7). Data are expressed as mean ± SEM. *p < 0.05 and **p < 0.01 by one-way analysis of variance with Tukey’s multiple comparisons test

The mean number of relative lung metastases was shown to be tenfold lower in mice that received 4T1.2-shOSM1 cells and fivefold lower in mice injected with 4T1.2-shOSM2 cells compared with 4T1.2-LacZ control cells (Fig. 4a). Histology performed on tissues from mice injected with parental 4T1.2 cells using an antimouse OSM antibody showed strong OSM expression in the primary mammary tumor, as well as some background expression in the normal breast connective tissue (Additional file 5: Figure S4). Specifically, very high OSM expression was seen at the leading edge of the primary mammary tumor, in closest proximity to the breast stroma.

MRI was used to track lung metastasis progression in vivo after injection of parental 4T1.2, control 4T1.2-shLacZ, and 4T1.2-shOSM2 cells. Mice were scanned postinjection at days 20–21, days 25–26, and just before being killed at days 29–30 (Fig. 4b). For all three cell lines, MRI studies showed essentially no detectable metastasis at days 20–21. However, at 25–26 days and 29–30 days, readily identifiable metastases were observed in lung images. The average metastasis volume was significantly decreased, by 50–80%, in 4T1.2-shOSM2 cells compared with 4T1.2-shLacZ control or parental 4T1.2 cells, respectively (Fig. 4c). There were also significant differences between the parental 4T1.2 cells and the control 4T1.2-shLacZ cells, which may be due to the potential off-target effects of shRNA activation [40–42]. This highlights the importance of using a true nontargeting shRNA control such as the 4T1.2-shLacZ cells because shRNA activation alone appears to have cell-static effects in cancer cells [40].

Further analysis revealed that the total number of metastases was also reduced by more than 50% in mice injected with 4T1.2-shOSM2 cells compared with control 4T1.2-shLacZ cells at 25–26 days and 29–30 days (Fig. 4d). Thus, in vivo MRI confirmed that OSM may be a potent inducer of the metastatic cascade that results in lung metastases originating from a primary mammary tumor. In sum, these results suggest that OSM is necessary for spontaneous mammary tumor metastasis to the lung in a syngeneic mouse model.

### Suppression of OSM by shRNA increases survival from spontaneous metastasis via orthotopic injection but not via intracardiac injection in vivo

**Fig. 5:**
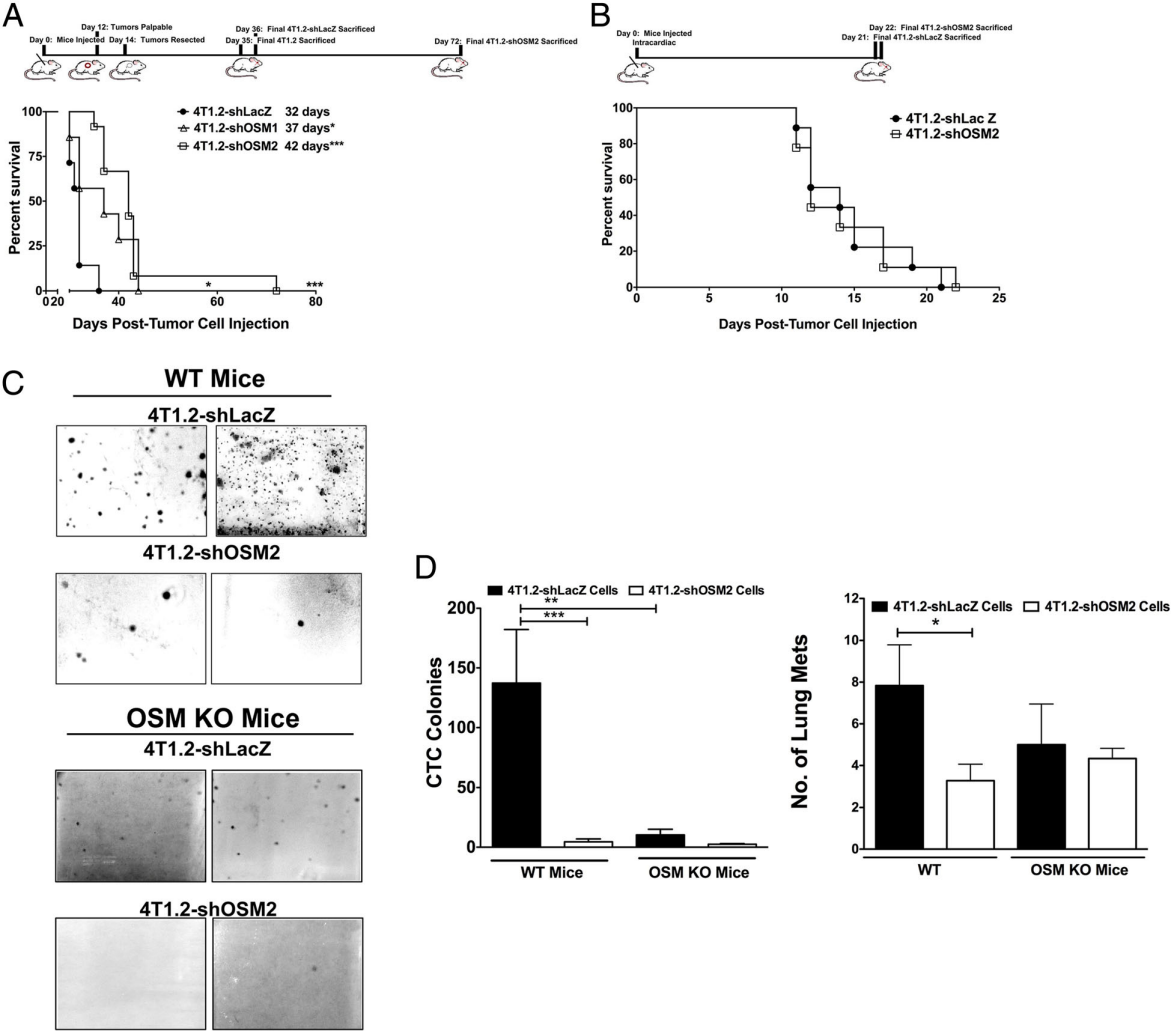
Reduced tumor cell oncostatin M (OSM) expression increases survival in a 4T1.2-shOSM mouse model of tumor resection. **a** Timeline shows orthotopic mouse mammary tumor cell injection at day 0, resection at day 14, and final day of killing per group (ranging from 35 to 72 days). Kaplan-Meier survival analysis following tumor resection showed that mice bearing 4T1.2-shOSM1 or 4T1.2-shOSM2 tumors had significantly increased survival compared with mice with control 4T1.2-shLacZ tumors. *p < 0.05 by log-rank test. **b** Timeline shows intracardiac mammary tumor cell injection at day 0 and final day of killing (days 21 to 22). Kaplan-Meier survival analysis showed no difference in survival between mice injected with control 4T1.2-shLacZ and those injected with 4T1.2-shOSM2 cells. **c** Blood was collected from wild-type and OSM-knockout (OSM-KO) animals with 4T1.2-shOSM2 or control tumors, and circulating tumor cell (CTC) counts were assessed via a colony-forming assay. Representative image depicts higher numbers of colonies that formed from the blood collected from wild-type mice with control tumors. **d** *Left*: Quantification of the colony-forming assay showed that wild-type animals bearing 4T1.2-shOSM2 tumors had a 15-fold lower number of CTCs than the animals bearing control 4T1.2-shLacZ tumors. Furthermore, OSM-KO mice with 4T1.2-shLacZ tumors had 10-fold less CTCs than wild-type mice bearing the same cells. *Right*: Wild-type mice bearing 4T1.2-shOSM2 tumors had a 2.5-fold lower number of lung metastases than mice with control 4T1.2-shLacZ tumors. OSM-KO mice bearing 4T1.2-shLacZ or 4T1.2-shOSM2 tumors had 2- to 2.5-fold less lung metastases than wild-type mice (4T1.2-shLacZ, *n* = 8–9; 4T1.2-shOSM1, *n* = 7; 4T1.2-shOSM2, *n* = 9–12). Data are expressed as mean ± SEM. *p < 0.05 and ***p < 0.001 by one-way analysis of variance with Tukey’s multiple comparisons test

We used a tumor resection survival model to mimic surgical removal of the primary tumor in patients and to determine if suppression of tumor-produced OSM limits early metastases. Orthotopic mammary fat pad injections were performed using control 4T1.2-shLacZ cells, 4T1.2-shOSM1 cells, and 4T1.2-shOSM2 cells. Primary tumors were resected when they became palpable at day 14 (Fig. 5a), and mice were monitored until endpoint criteria were met (*see* the Methods section above). The mean survival time of the mice that received 4T1.2-shOSM1 and 4T1.2-shOSM2 cell injections significantly increased, by 5 and 10 days, respectively, compared with animals with 4T1.2-shLacZ tumors (Fig. 5a). These results suggest that following primary mammary tumor resection, decreased OSM expression in primary tumor cells leads to increased survival.

In order to determine if OSM affects postintravasation aspects of metastasis, we injected the mammary tumor cells directly into the circulatory system via the left ventricle of the heart. There was no statistical difference in the survival time between mice injected intracardially with 4T1.2-shLacZ versus 4T1.2-shOSM2 cells (Fig. 5b). Similarly, there was no statistical difference in lung metastatic burden in the two different tumor types as assessed by qPCR (Additional file 6: Figure S5). These results suggest that tumor cell OSM expression has little effect on the postintravasation aspects of metastasis to lung, such as extravasation and metastatic site implantation.

### CTC number and metastatic burden is reduced in OSM-knockout mice compared with wild-type mice

To determine if knocking out OSM in the whole organism affects CTC numbers in the 4T1.2 mouse model, wild-type and OSM-KO BALB/c mice were orthotopically injected with either 4T1.2-shLacZ or 4T1.2-shOSM2 cells. Whole blood was collected at the endpoint, RBCs were lysed, and the remaining white blood cells containing the epithelial CTCs were examined using a clonogenic assay (Fig. 5c). Blood from control mice with no tumors had no colony formation (Additional file 7: Figure S6). Blood from OSM-KO mice injected with 4T1.2-shOSM2 cells had 15-fold fewer CTCs and 2.5-fold fewer lung metastases than wild-type mice injected with control 4T1.2-shLacZ cells (Fig. 5d, left). Additionally, OSM-KO mice bearing 4T1.2-shLacZ tumors had 10-fold fewer CTCs and 2.5-fold fewer lung metastases than wild-type mice with the same tumor type (Fig. 5d, right). In a separate in vitro assay to test the level of colony formation, there were no differences in colony numbers between the cell lines when seeded at low numbers (~ 10 cells) (Additional file 8: Figure S7), which indicates that the OSM-knockdown cells do not have reduced ability to survive and develop colonies. These results suggest that microenvironment OSM, independent of tumor cell-secreted OSM, has a large effect on tumor cell dissemination into the circulation. Furthermore, this highlights the importance of paracrine OSM in breast cancer progression and metastasis.

### OSM increases preintravasation metrics of metastatic capacity in 4T1.2 cells

**Fig. 6:**
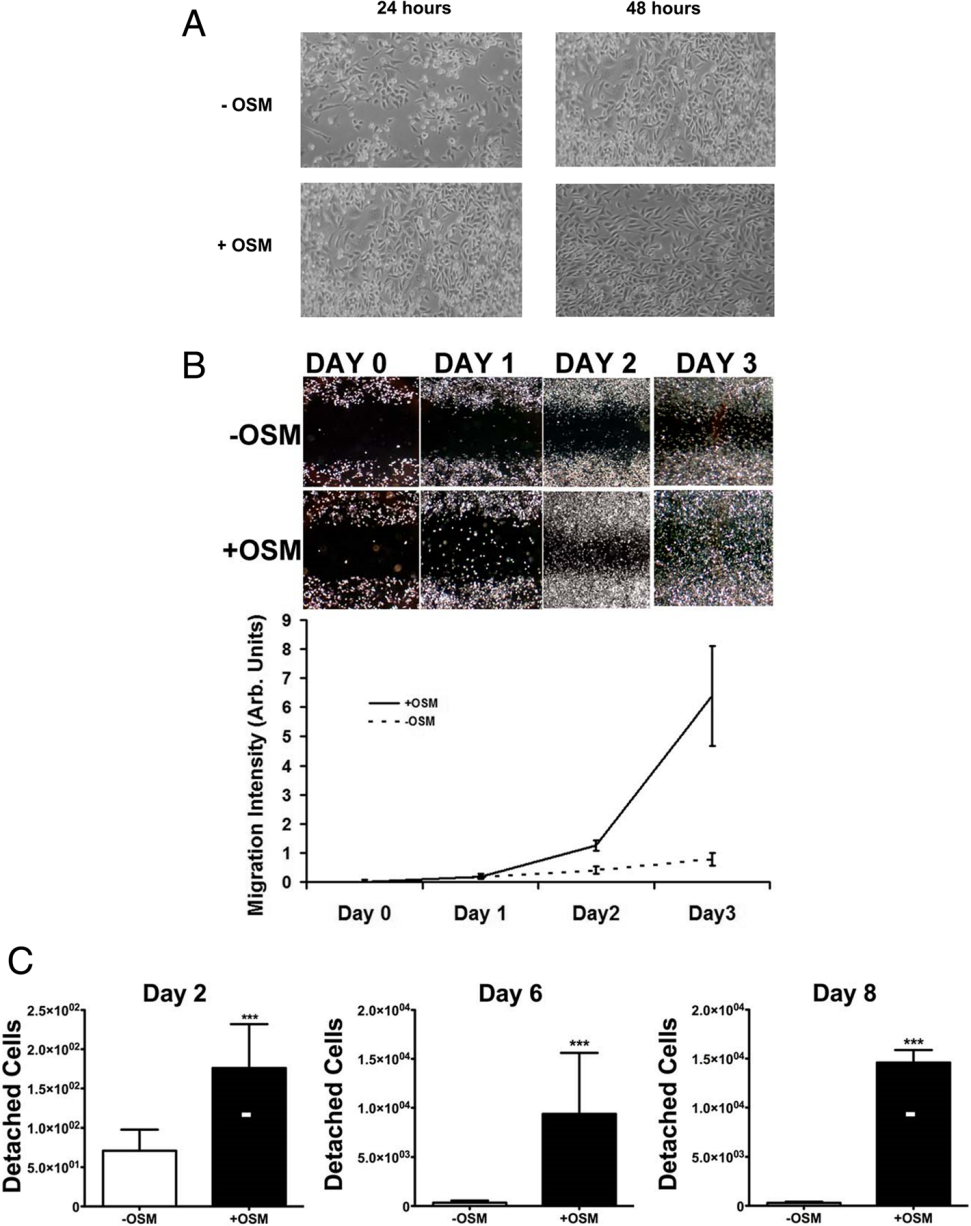
Oncostatin M (OSM) promotes 4T1.2 cell detachment and migration. **a** 4T1.2 mouse mammary cancer cells were plated and treated with recombinant murine OSM (25 ng/ml) for 24 and 48 hours. No morphological changes indicative of epithelial-mesenchymal transition were detected. **b** 4T1.2 cells were grown to 80% confluence, and a uniform scratch was made. Cells treated with OSM had higher levels of migration than untreated controls (sevenfold by day 3). **c** A detachment assay was performed on 4T1.2 cells, and the number of detached cells was quantified. Cells treated with OSM had significantly higher numbers of detached cells (100-fold at day 8). Data are expressed as mean ± SEM. *p < 0.05 and ***p < 0.001 by two-tailed Student’s *t* test

For tumor cells to enter the bloodstream as CTCs and subsequently metastasize, it is thought that they must first undergo EMT, followed by detachment, migration, and intravasation into the circulatory system [43]. Although OSM has been shown to increase tumor cell detachment and migration and to induce EMT in human breast cancer cell lines [16, 20–22, 44], no data have been published on murine 4T1.2 cells in relation of OSM’s in vitro effects. TNBC (ER−, PR−, HER2−) 4T1.2 mouse mammary cancer cells were treated with OSM (25 ng/ml) for 24 to 48 hours. On one hand, because 4T1.2 cells are an aggressive mesenchymal mammary cancer cell type, OSM did not affect cell morphology or produce an EMT in vitro (Fig. 6a). On the other hand, OSM significantly increased 4T1.2 mammary tumor cell migration 7-fold by day 3 in a cell migration assay (Fig. 6b) and tumor cell detachment 100-fold by day 8 (Fig. 6c). Our previous studies also demonstrated that OSM increases overall invasive potential in 4T1.2 cells [7]. These increases in migration and detachment were seen despite a 20% inhibition by OSM on 4T1.2 cell proliferation (Additional file 9: Figure S8). Together, these results suggest that OSM may promote tumor cell dissemination into the circulation by increasing cell migration and detachment, which may subsequently increase the number of CTCs.

## Discussion

**Fig. 7:**
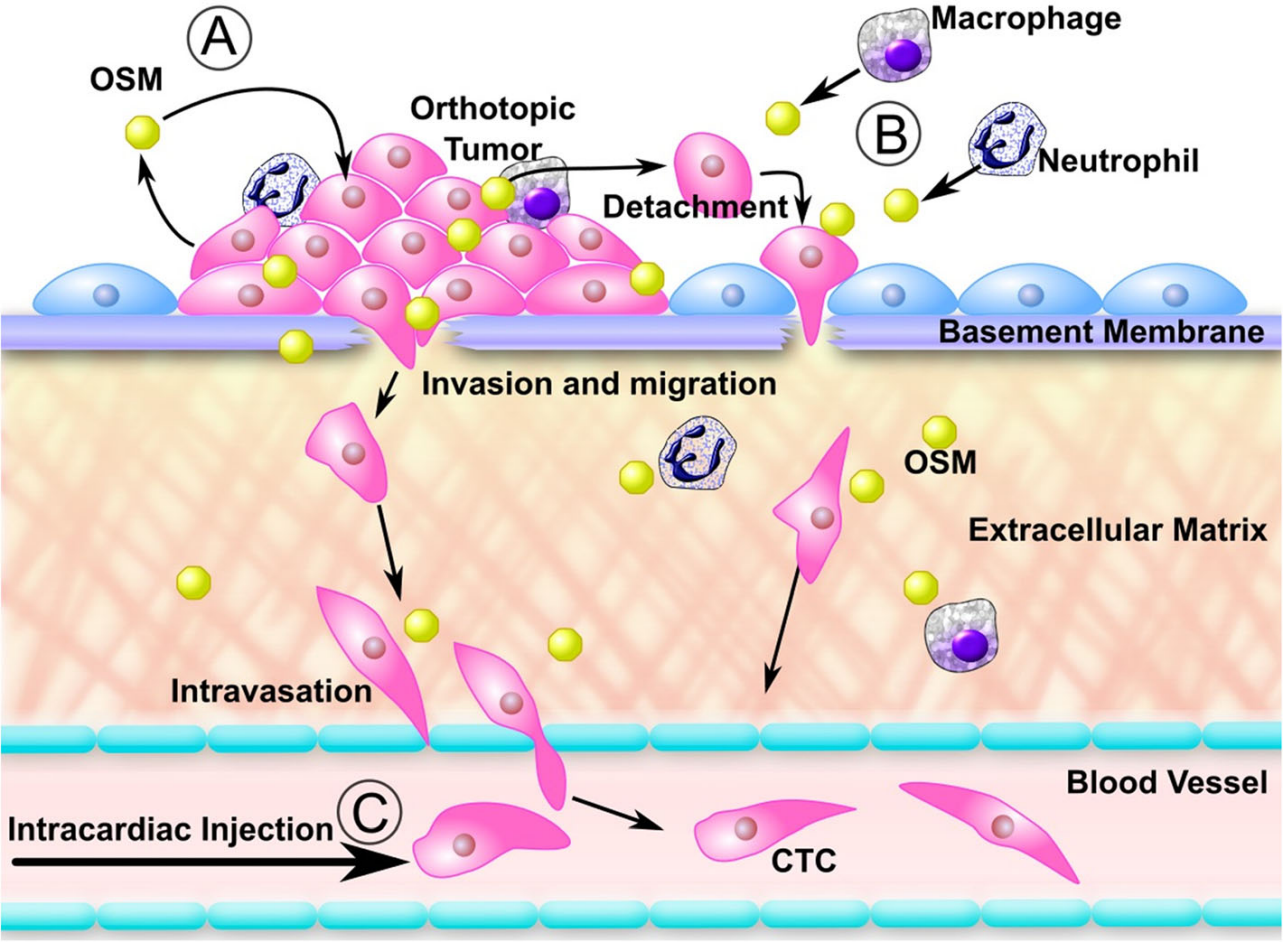
Model of oncostatin M (OSM)-mediated metastasis. OSM is produced in an autocrine fashion by tumor cells (**a**), as well as by tumor-associated macrophages and neutrophils (**b**), for paracrine signaling. OSM promotes preintravasation effects, such as tumor cell detachment and migration, that can drive tumor cell intravasation into the circulation to develop circulating tumor cells and eventually metastasis. **c** When tumor cells are injected directly into the circulatory system, they bypass the multistep preintravasation aspects of metastases, and our data suggest that OSM has little effect on their extravasation and colonization at a secondary site

In this paper, we show that OSM, whether acting in a paracrine fashion or produced by breast tumor cells and acting in an autocrine manner, can potentiate preintravasation metastatic events, such as migration, detachment, and increased CTCs (Fig. 7). Recent studies suggest that cells from DCIS can actually metastasize prior to their development into malignant IDC, though what triggers this early event has not been well characterized [45]. Our breast cancer IHC studies using TMAs resulted in an intriguing finding: OSM expression is highest in the epithelium of DCIS, as compared with IDC, metastatic, or adjacent normal tissue, though OSM levels in IDC are also high. When looking at the adjacent stroma, we found reduced levels of OSM in the fibroblasts and blood vessels of IDC tissues compared with normal tissue. On one hand, this is interesting because we have previously shown that secreted OSM binds to proteins of the ECM and bioaccumulates in the tumor’s acidic microenvironment [11]. On the other hand, García-Tuñón et al. observed higher levels of breast tissue OSM and OSMR in IDC than in DCIS or normal tissue [8]. Although this finding differs somewhat from our study in relation to the stage at which the highest level of OSM was seen, there is agreement that higher OSM levels were detected in cancerous tissue than in normal tissue.

In this study, three different TNBC mouse models were used: One was an immunocompetent BALB/c model using syngeneic 4T1.2 cells, and two were immunosuppressed athymic xenograft mouse models, using either MDA-MB-231-Luc2 or MDA^TO/OSM^ cells. Despite the differences between the systems used in our study, our results were consistent in that suppression of OSM reduced metastasis in BALB/c mice and injection of recombinant hOSM or TET-induced hOSM expression in MDA^TO/OSM^ cells increased human breast tumor metastasis in athymic mice. Although adaptive immunity is stunted in athymic mice owing to nonfunctional T cells, innate immune function is still intact [46]. Recent studies indicate that innate immunity plays a primary role in controlling progression of tumor growth and metastatic disease [47], and innate immune cells such as macrophages home to hypoxic tumors and promote angiogenesis [10]*.* OSM has recently been shown to increase the tissue infiltration of both the proinflammatory M1 and the wound-healing M2 macrophages [48]. Specifically, Lauber et al. demonstrated that OSM promotes lung metastatic burden in melanoma through M2 macrophage infiltration [49]. Thus, OSM may be promoting prometastatic responses, mediated by innate immunity, in the tumor microenvironment of both athymic and BALB/c mouse models to promote metastases.

Although the traditional cause of mortality in patients with advanced cancer is metastasis to vital organs, cachexia has been shown to contribute up to 50% of cancer patient deaths [50]. Significant weight loss as a consequence of fat loss and muscle wasting, indicative of cachexia, was seen in our TET-induced OSM in MDA^TO/OSM^ xenograft mouse model. TET alone does not appear to have any effect on kidney function [51]. Interestingly, TET has been shown to actually reduce tumor cell growth and aggressiveness [52], but this effect was not seen in our studies. Other studies have shown that high levels of various inflammatory cytokines, such as IL-6, potentiate loss of adipose tissue and muscle wasting [53]. Therefore, OSM may be yet another cytokine that could exacerbate cachexia in patients with breast cancer. In this study, mice with 4T1.2-shOSM1 tumors had the lowest lung metastatic burden despite the fact that 4T1.2-shOSM2 cells have lower OSM expression [7]. However, this reduced lung metastasis in 4T1.2-shOSM1 tumor-bearing mice did not translate to increased survival for 4T1.2-shOSM2 tumor-bearing mice. This supports the notion that metastatic burden may not be the only cause of increased mortality, but that elevated cytokine levels may contribute to reduced survival. Furthermore, because cytokines modulate the immune system, it is very probable that cancer cachexia is related to maladaptive immune responses [54]. Interestingly, there were also major differences between the parental 4T1.2 cells and the control 4T1.2-shLacZ cells, where the shLacZ cells had significantly reduced lung metastasis volume. This highlights the importance of using a nontargeting shRNA control cells because shRNA activation has been known to have multiple off-target effects leading to reduced cell survival, metastasis, and growth [40–42, 55].

Platelets, as an adjunct to their classical role in thrombosis, are also important in mediating inflammation and immune response [56]. We found that in the MDA^TO/OSM^ mouse model, higher OSM levels were correlated with increased platelet counts and reduced animal survival. Previous studies in patients with breast cancer have shown that elevated platelet counts were associated with poor prognosis and reduced disease-free survival [57]. Platelets have also been implicated in the promotion of metastasis by acting as a reservoir for factors that induce invasion and function to protect CTCs from the immune system [58]. In our study, increased viable CTCs were detected in our mouse models when higher levels of OSM were present, which may have been due to the elevated levels of platelets in circulation.

The ability of tumor cells to intravasate into the circulation directly correlates with CTC numbers from the corresponding tumor [59]. Increased CTC numbers have been linked clinically to enhanced metastatic burden in patients and a reduced 5-year survival rate [60]. Because tumors may shed early during cancer development [61], the detection of CTCs would be an important tool in the clinic to assess the metastatic capacity of a tumor, even as early as in precancerous DCIS. Typically, CTCs are detected using cancer epithelial markers, such as cytokeratins 18/19 [62]. However, highly aggressive tumor cells that have already undergone EMT, and thus have lost their epithelial markers, may evade detection [63]. Other possibly viable markers for CTC detection include epithelial cell adhesion molecule and human mammaglobin A [62]. For our studies, the highly aggressive mesenchymal-like MDA-MB-231 cells show negative or low expression for each of these markers, making conventional CTC detection unfeasible [63]. Thus, we employed multiple techniques that are marker-independent, such as the colony-forming assay for the mouse mammary tumor model or a PCR assay targeting human Alu sequences in the human breast tumor model. We did not use the colony-forming assays to assess the human breast tumor model, because the MDA-MB-231 cells have a highly variable in vitro survival rate when seeded at low numbers. 4T1.2 cells showed no difference in the overall level of cell survival when seeded at low numbers in vitro, which makes the colony-forming assay ideal for the 4T1.2 tumor model.

In our studies, suppression of tumor-produced OSM or the absence of OSM in OSM-KO mice resulted in reduced numbers of CTCs, whereas injection of recombinant OSM increased CTCs. There was no significant difference in the number of CTCs with or without TET in the MDA^TO/OSM^ mouse metastasis model (data not shown). This suggests that paracrine OSM may be more important than autocrine-produced OSM for CTC development. Indeed, in our OSM-KO mouse model, where there is less paracrine OSM, total CTC numbers and lung metastatic burden were significantly reduced compared with WT mice. Although the number of CTCs appeared modest in our study, these numbers are actually higher than the low numbers of CTCs seen in previously published studies [64, 65]. It has been reported that even in advanced cancers, there can be as few as 1–5 CTCs per 7.5 ml [66]. Our higher CTC numbers may point to differences in human and mouse physiology and may explain the much more rapid progression of metastatic disease seen in mice than in patients.

To assess some of the early aspects of metastasis that could lead to generation of CTCs in vitro, tumor cell EMT, migration, and detachment were studied in the highly aggressive 4T1.2 tumor model. 4T1.2 mammary tumor cells, which are considered analogues of high-grade human TNBC, have already undergone EMT, and OSM did not cause additional EMT-like effects [67]. However, our results show that OSM does increase cell migration, detachment, and invasion in 4T1.2 cells [7], supporting the idea that OSM operates in the preintravasation steps of metastasis. Other OSM-related factors, such as IL-6 and IL-8, may promote CTCs by increasing tumor cell invasion, detachment, and EMT [68]. Furthermore, these effects may be amplified in vivo because OSM possesses a proclivity to accumulate in the acidic ECM of the tumor microenvironment [11].

On the basis of our findings, OSM may potentiate the preintravasation aspects of the metastatic cascade to increase metastasis to the lung and bone [7]. This is evidenced by the fact that intracardiac injection of 4T1.2 cells with reduced OSM (4T1.2-shOSM2) into mice, which bypasses the intravasation step of the metastatic cascade, did not result in increased survival compared with control cells (4T1.2-shLacZ). Therefore, it is highly probable that OSM functions before intravasation during cancer progression and suggests that OSM does not affect CTC survival, tumor cell extravasation, and/or secondary tumor growth.

## Conclusions

The results of this study suggest that OSM increases lung metastases and CTC numbers by acting on early metastatic events (Fig. 7). Specifically, OSM increases tumor cell migration, detachment, and invasion [7], supporting the idea that OSM operates in the preintravasation steps of metastasis. Inhibition of OSM and/or OSMR has demonstrated antitumor effects and has recently been receiving increased attention as a possible cancer therapy [69, 70]. However, to our knowledge, no small-molecule inhibitors or humanized antibodies that target OSM signaling are in clinical trials for metastatic breast cancer. Taken together, the findings of this study provide a rationale for the administration of anti-OSM therapeutics before tumor resection, at the earlier stages of the disease, when the potential to improve overall survival of patients with breast cancer is greatest.

## Additional files

Additional file 1: **Table S1.** Primer and probe sequences used for qPCR assay for the detection of CTC in blood obtained from tumor-bearing mouse. **Table S2.** Table format data for Fig. 1B show total number of patients and cores for each stage of breast tissue assessed. **Table S3.** Mean expression levels are statistically significantly different among cancerous and normal tissues for both stroma and blood vessel endothelium (*p*<.001). **Table S4.** Margin status, Her2/neu status and estrogen receptor (ER) status, were revealed by repeated measures analysis. Mean expression levels are statistically significantly different among cancerous, normal, and metastatic tissues for margin status, and ER status. For Her2 status, significant differences were found among cancerous, normal and between 0 and 1 staining intensity for metastatic tissues (*p*<.001). (DOCX 343 kb)

Additional file 2: **Figure S1.** qPCR standard curve derived from spiking cancer cells into mouse blood. MDA-MB-231 cells were spiked into mouse blood, and DNA was extracted and subjected to qPCR analysis. Specific cell numbers were correlated to CT values and were used to construct a standard curve for the CT values extrapolated from experimental mouse blood. (PPTX 186 kb)

Additional file 3: **Figure S2.** Representative OSM staining intensity in IHC. 0 = no staining (image not shown), 1 = light staining, 2 = moderate staining, 3 = heavy staining. (PPTX 27 kb)

Additional file 4: **Figure S3.** Deterioration of physical condition in MDA^TO/OSM^ tumor-bearing mice treated with TET. **a** MDA^TO/OSM^ tumor-bearing mice treated with tetracycline (+TET) lost, on average, 11.4% of their body weight during TET treatment, compared with −TET mice, which gained an average of 5.5% of their body weight over the same period. **b** Representative image of mice with MDA^TO/OSM^ tumors +TET shows prominent spinal column, muscle wasting, and lack of visible adipose tissue. **c** Gross morphology of normal (left) and abnormal kidneys (right). Normal kidneys have a distinct border between the medulla and the cortex, with the cortex shown in a darker pink/red color and the medulla shown in a lighter pink color. This indicates that normal blood perfusion was taking place. Abnormal kidneys were either both pale and hypoperfused (middle) or damaged (right), with no clear distinction between the cortex and the medulla. **d** One hundred percent of mice in the +TET group have abnormal kidney morphologies, whereas only 25% of the mice in the −TET group have abnormal kidneys. **p < 0.01 by Fisher’s exact test. **e** Sera from mice with abnormal kidneys have a statistically significant higher level of OSM than sera from mice with normal kidneys. Data are expressed as mean ± SEM. *p < 0.05, **p < 0.01, and ***p < 0.001 by two-tailed Student’s *t* test. (ZIP 135 kb)

Additional file 5: **Figure S4.** OSM is highly expressed in orthotopic 4T1.2 primary mammary tumors in female BALB/c mice. Histology using H&E confirmed the presence of a large primary mammary tumor (T) 32 days after 4T1.2 mouse mammary tumor cell injection into the fourth mammary fat pad of female BALB/c mice. High OSM expression is seen in the tumor, as is background expression in the normal breast connective tissue (CT). OSM expression is shown to be highest in the invasive edge of the tumor (T) closest to the normal breast connective tissue (CT). Control slides with no primary OSM antibody show low background staining. (PPTX 315 kb)

Additional file 6: **Figure S5.** qPCR analysis of lung metastases after intracardiac injections. 4T1.2-shLacZ cells and 4T1.2-shOSM2 cells were introduced via intracardiac injection, and qPCR analysis of the lung metastases indicated that the difference between the groups was not significant by two-tailed Student’s *t* test. (ZIP 60 kb)

Additional file 7: **Figure S6.** Control colony-forming assay results derived from non-tumor-bearing mice. Blood from non-tumor-bearing mice contained no cells that formed colonies. (PPTX 53 kb)

Additional file 8: **Figure S7.** Test of cell line-specific variance in colony-forming assay between 4T1.2-shLacZ and 4T1.2-shOSM2 cell lines. Approximately 10 and 50 cells of 4T1.2-shLacZ or 4T1.2-shOSM2 cells were seeded onto tissue culture plates and were allowed to incubate until colony formation. No significant differences between the cells were detected with ~ 10 cells seeded; however, there was a small but significant increase in the number of colonies with 4T1.2-shOSM2 cells at 50 cells seeded. Data are expressed as mean ± SEM. *p < 0.05 by one-way ANOVA with Bonferroni’s multiple comparisons test. (PPTX 68 kb)

Additional file 9: **Figure S8.** OSM inhibits proliferation of 4T1.2 cells. One hundred 4T1.2 cells were plated at day 0 and treated with 25 ng/ml of OSM. By day 7, there was a 20% reduction in total cell numbers in the OSM-treated group versus the non-OSM-treated group. Data are expressed as mean ± SEM. *p < 0.05, **p < 0.01; statistical analysis was performed for each day using a two-tailed Student’s *t* test. (PPTX 21 kb)
