# Supplementary material for: OSM potentiates preintravasation events, increases CTC counts, and promotes breast cancer metastasis to the lung
Source: Breast Cancer Res. 2018 Jun 14;20:53. doi: 10.1186/s13058-018-0971-5 (PMC6001163; doi:10.1186/s13058-018-0971-5)

**Supplemental Materials and Methods**

**Complete details for tissue microarrays.** Breast tissue was obtained from paraffin block archives at the Department of Pathology, Mercy Medical Center, Nampa, ID and de- identified, as per IRB guidelines. Three tissue microarrays (TMA) of 1-µm thickness and totaling 72 patients were made using a Quick-Ray, an instrument used for boring tissue from a paraffin block (Woo-Ri Medic, Kent, WA). The TMAs were built in Dr. William Fyffe’s lab at Northwest Nazarene University, Nampa, ID. Two blocks consisted of tissues from 54 breast cancer patients (32 adjacent normal, 9 DCIS patients, 54 IDC) without metastasis, and included three primary tumor cores and one adjacent normal core for each case. The third block included samples from a total of 18 breast cancer patients (18 adjacent normal, 3 DCIS, 18 IDC and 16 metastatic) with lymph node metastasis and contained three primary tumor cores, two metastatic cores and one adjacent normal tissue core per case. The TMAs included a row of control tissues including spleen, lung, placenta, salivary gland, liver and brain. Spleen and salivary gland served as positive controls for OSM staining.

Immunohistochemistry: The TMAs were stained for oncostatin M using the Histostain Kit (Invitrogen, Carlsbad, CA; Cat #95-9843) per manufacturer’s instructions. The TMAs were deparafinized using Histosol (National Diagnostics, Atlanta, GA) and stained overnight with 1:400 dilution of rabbit anti-human OSM primary antibody (Santa Cruz Biotechnology, Santa Cruz, CA; Cat #sc-129) and 1 hour with 1:1000 goat-anti rabbit IgG-AP secondary antibody. TMAs stained with secondary antibody alone served as the negative control, and spleen and salivary gland served as positive controls for OSM staining. The specificity of the ɑ-OSM antibody was tested by treating tissue sections with 10 times the amount of OSM blocking peptide (Santa Cruz Biotechnologies, Santa Cruz, CA) and incubating overnight at 4^0^C. The mixture was then diluted to the required concentration and immunohistochemistry was performed, as above.

The TMAs were analyzed for OSM expression independent of cell density by an experienced pathologist, Dr. Joseph Kronz, at Mercy Medical Center, Nampa, ID. The intensity of OSM staining in the ductal epithelium was graded as follows: 0=No staining; 1=Light staining; 2=Medium staining; 3=Dark staining. To confirm reproducibility of the results, the pathologist, Dr. Kronz, reread ten TMA cores that were chosen at random, and his observations were consistent with the previous results. In cases where a single core had both cancerous and normal tissue, the OSM expression data in the cancerous part was combined with other cancerous tissues. Similarly, the expression in the normal part was combined with other adjacent normal tissues for statistical analysis.

Statistical analysis: Assessments from multiple cores for each patient were averaged for epithelial cells and stage of malignancy (normal, DCIS, IDC, metastatic) that was present in the core. These four stages were statistically compared among ductal tissues. The OSM staining intensity was analyzed as a mixed model to accommodate repeated observations on each patient. These repeated observations were assumed to have non-negligible correlation and were modeled under standard repeated measures variance-covariance assumptions. Stage was treated as a fixed effect. Additionally, statistical models considering patient prognostic markers were evaluated. These models included the stage and the prognostic marker, with or without an interaction. The model with the lowest AIC_c_ was selected to determine whether the prognostic factor was associated with OSM staining. Prognostic factors considered were age, tumor size, lymph node status, angiolymphatic invasion, tumor grade, tumor type, histologic grade, nuclear atypia, margin status, mitotic rate, Her2/neu expression, progesterone, and estrogen receptor. Initial assessments indicated that OSM staining intensity did not differ significantly between the two groups of patients (those with and without lymph node metastasis) so patient group was not included as an analysis factor in the study. All models were assessed for adequacy by residual analysis, a concern here because of the bounds on OSM staining intensity (0-3) and our specific interest in changes in mean staining intensities. No predicted values exceeded the possible observational boundaries and residual patterns were acceptable despite the categorical nature of the data collection. All analyses were conducted using SAS version 9.1.3 (SAS 2004).

**Animal Histology**

Mammary tumors were (4T1.2-LacZ, n=2; 4T1.2-shOSM2, n=2; and 4T1.2-shOSM1, n=1) were placed in ultralight fixative (Ultralight Histology, Nampa, ID) paraffin embedded, and sectioned (Bi-Biomics, Nampa, ID). The sections were then stained overnight with 1:400 dilution of goat anti-mouse OSM primary antibody (Cat # AF-495-NA; R&D systems) and for one hour with 1:1000 donkey-anti goat IgG-AP secondary antibody. The sections were then stained and imaged.

**Cell Proliferation Assay**

4T1.2 cells were plated on 24-well plates at a density of 100 cells per well in MEM-alpha media. OSM treated wells contained 25 ng/mL of rhOSM and the cell numbers were counted every other day.

**Supplemental Material**

**Table S1: qPCR primer and probe sequences.**


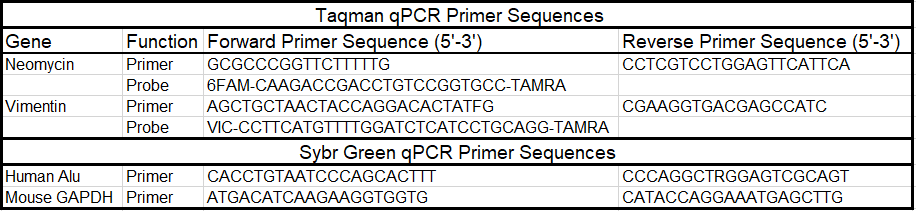


**Table S2: Comparison of OSM expression in the ductal epithelial cells of different stages of ductal carcinoma of the breast (DCIS, IDC and metastatic tissues) and adjacent normal breast tissues.** Mean expression levels are statistically significantly different among the four stages.

| **Stage** | **No. of patients (Total no. of cores)** | **Mean OSM staining** | **95% Confidence Limits** | **Pairwise comparison of stage means^1^** |
| --- | --- | --- | --- | --- |
| Adjacent Normal DCIS  IDC  Metastatic | 50 (83)  12 (18)  72 (188)  16 (29) | 1.33  2.00  1.66  1.24 | (1.15,1.50) (1.71,2.30) (1.55,1.77) (1.02,1.46) | A  B  B  A |
| ^1^Stages with the same letter are not statistically different; those with different letters are significantly different at p<0.05. | | | | |

**Table S3: Comparison of OSM expression in adjacent normal and cancerous stroma and blood vessel endothelium.** Mean expression levels are statistically significantly different among cancerous and normal tissues for both stroma and blood vessel endothelium (p<.001)


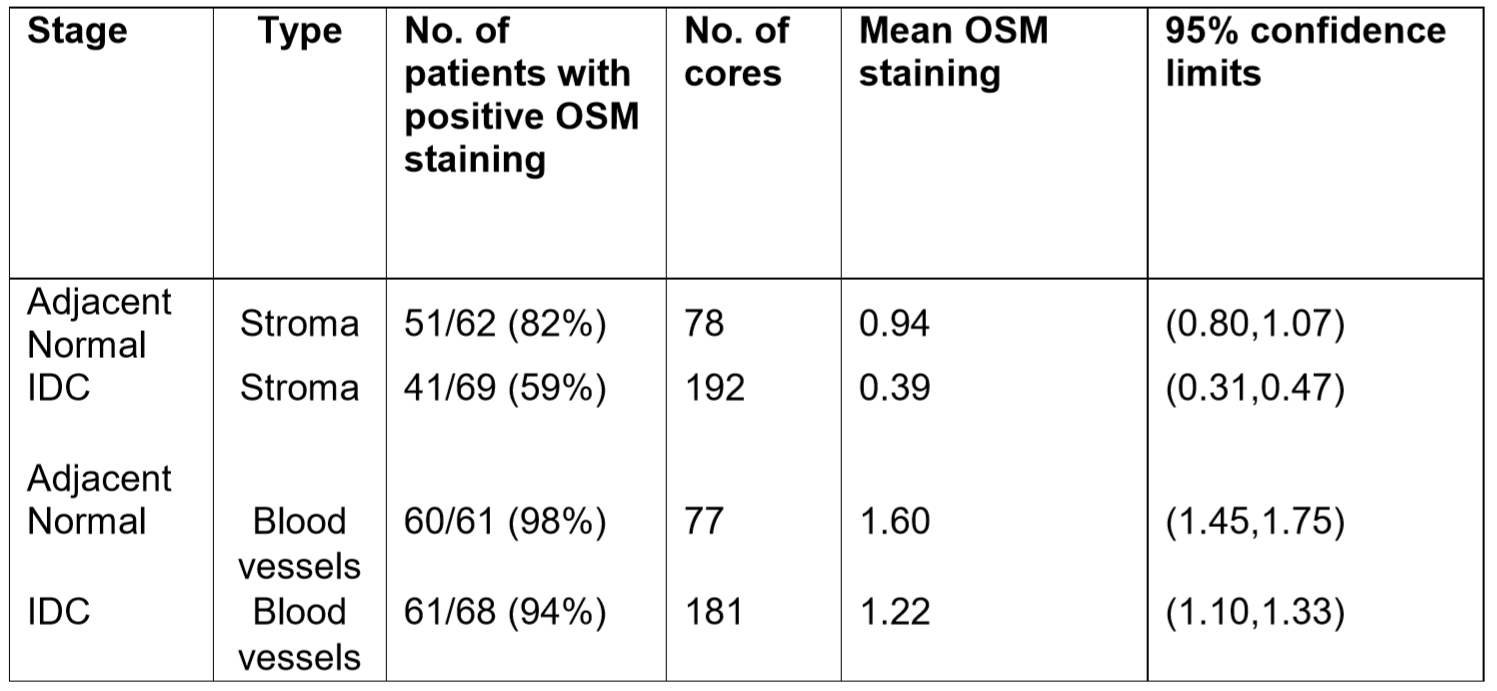


**Table S4: Comparison of OSM expression in correlation with breast cancer prognostic markers.** Margin status, Her2/neu status and estrogen receptor (ER) status, were revealed by repeated measures analysis. Mean expression levels are statistically significantly different among cancerous, normal, and metastatic tissues for margin status, and ER status. For Her2 status, significant differences were found among cancerous, normal and between 0 and 1 staining intensity for metastatic tissues. (p<.001)


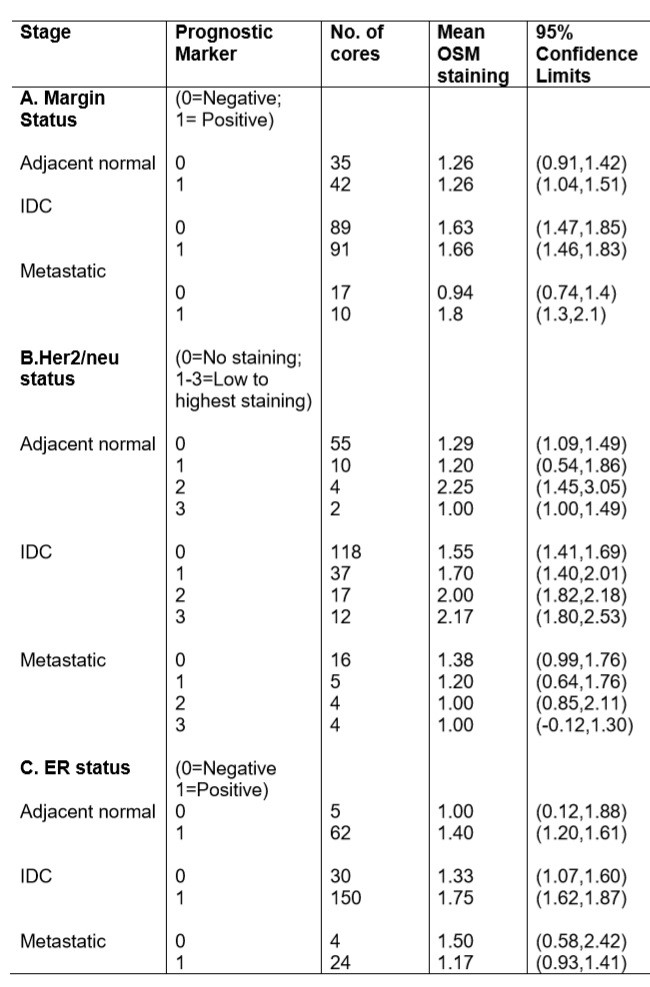

Supplement: Supplementary file 1 — Table S1. Primer and probe sequences used for qPCR assay for the detection of CTC in blood obtained from tumor-bearing mouse. Table S2. Table format data for Fig. 1B show total number of patients and cores for each stage of breast tissue assessed. Table S3. Mean expression levels are statistically significantly different among cancerous and normal tissues for both stroma and blood vessel endothelium (p<.001). Table S4. Margin status, Her2/neu status and estrogen receptor (ER) status, were revealed by repeated measures analysis. Mean expression levels are statistically significantly different among cancerous, normal, and metastatic tissues for margin status, and ER status. For Her2 status, significant differences were found among cancerous, normal and between 0 and 1 staining intensity for metastatic tissues (p<.001). (DOCX 343 kb) [file 13058_2018_971_MOESM1_ESM.docx]
