# Supplementary figures and images for: OSM potentiates preintravasation events, increases CTC counts, and promotes breast cancer metastasis to the lung
Source: Breast Cancer Res. 2018 Jun 14;20:53. doi: 10.1186/s13058-018-0971-5 (PMC6001163; doi:10.1186/s13058-018-0971-5)

## Slide 1
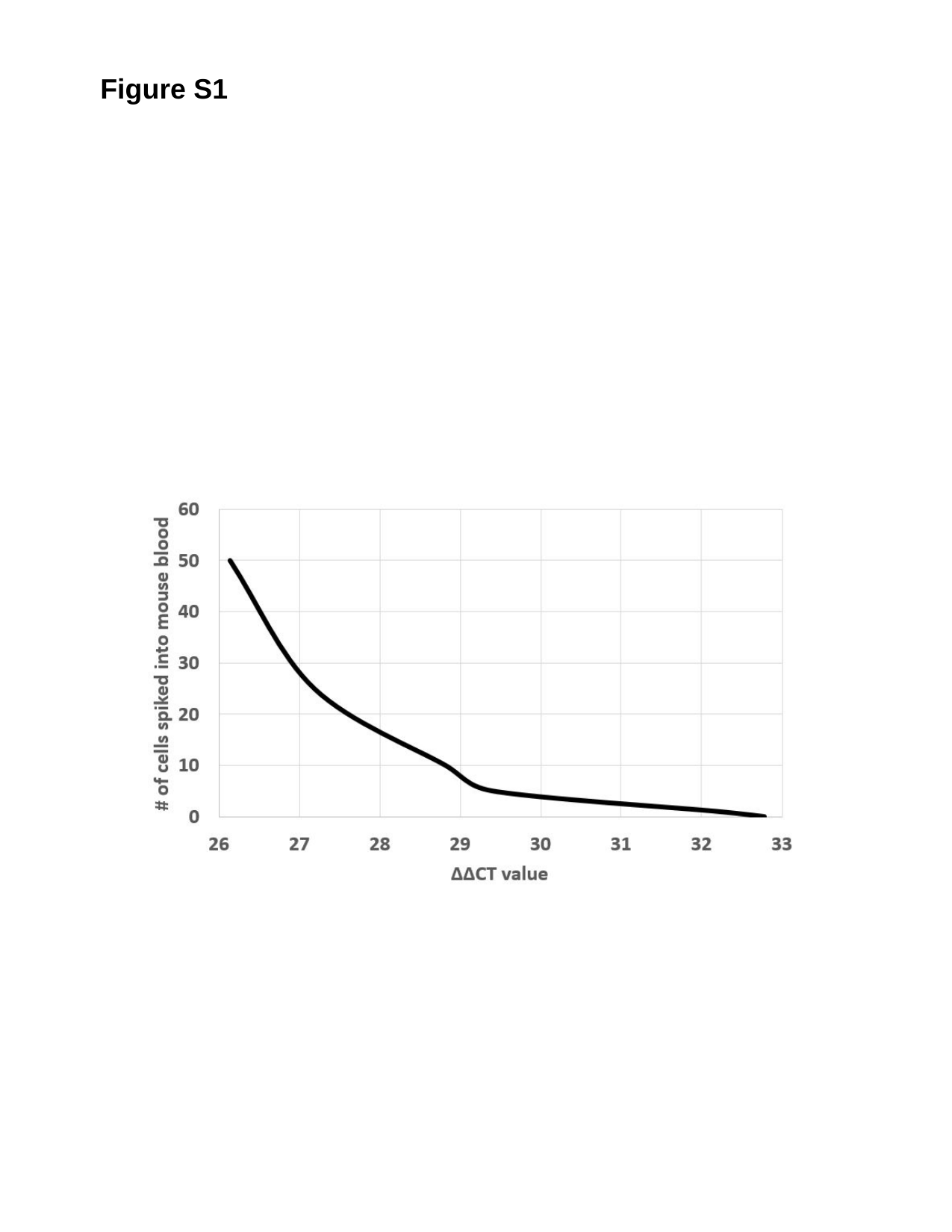

Figure S1

Supplement: Supplementary file 2 — Figure S1. qPCR standard curve derived from spiking cancer cells into mouse blood. MDA-MB-231 cells were spiked into mouse blood, and DNA was extracted and subjected to qPCR analysis. Specific cell numbers were correlated to CT values and were used to construct a standard curve for the CT values extrapolated from experimental mouse blood. (PPTX 186 kb) [file 13058_2018_971_MOESM2_ESM.pptx]

## Slide 1
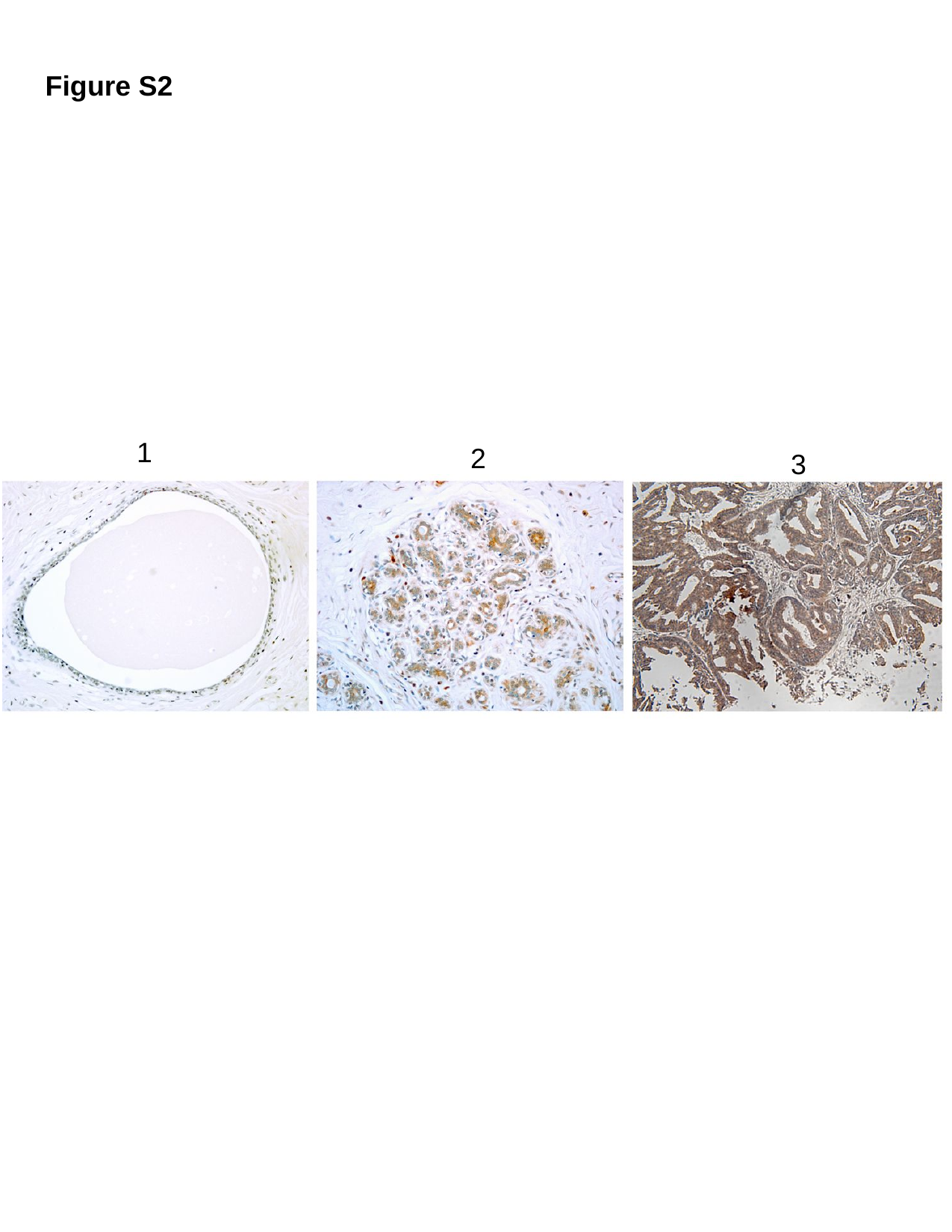

Figure S2
1
2
3

Supplement: Supplementary file 3 — Figure S2. Representative OSM staining intensity in IHC. 0 = no staining (image not shown), 1 = light staining, 2 = moderate staining, 3 = heavy staining. (PPTX 27 kb) [file 13058_2018_971_MOESM3_ESM.pptx]

## Slide 1
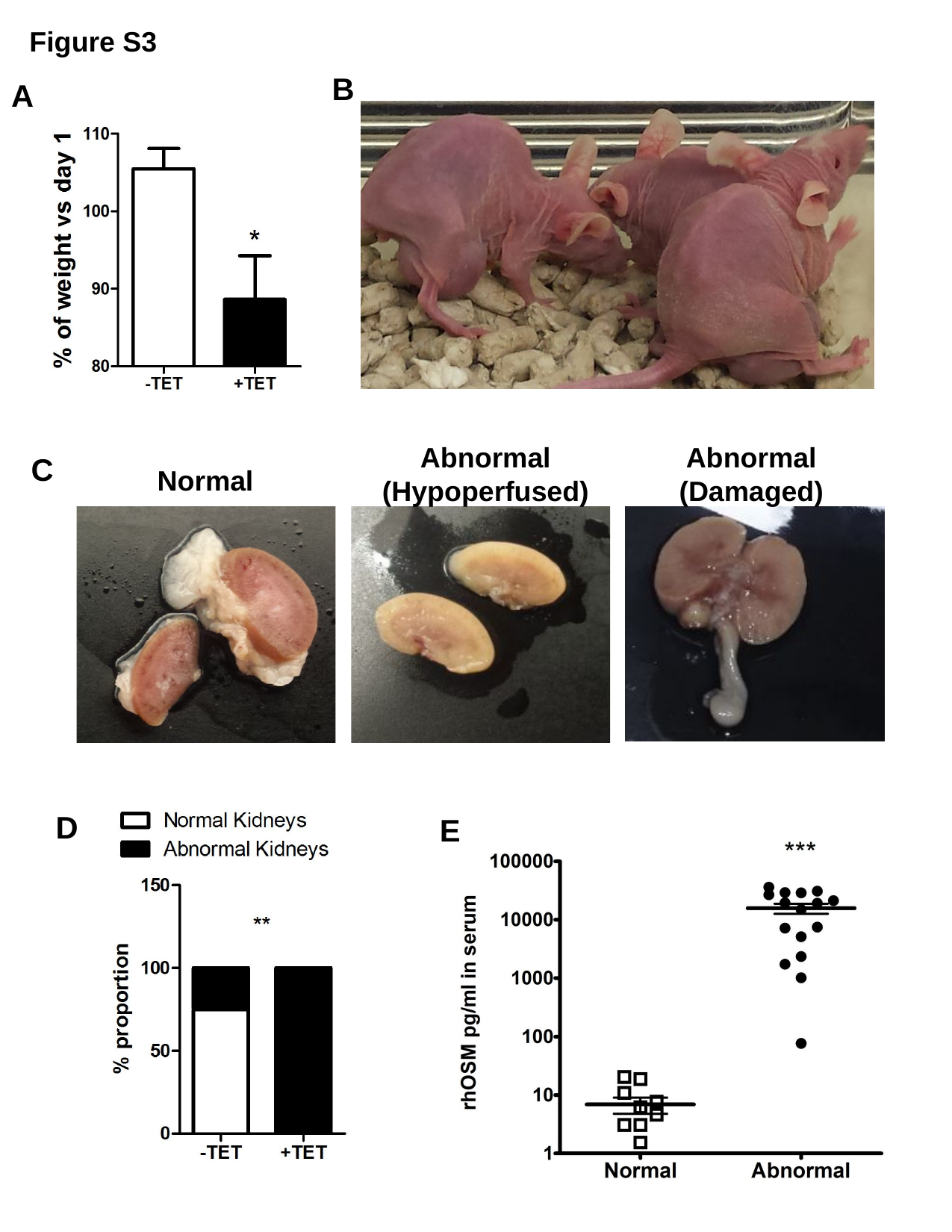

Figure S3
B
A
Abnormal
(Hypoperfused)
Abnormal
(Damaged)
C
Normal
D
E

Supplement: Supplementary file 4 — Figure S3. Deterioration of physical condition in MDATO/OSM tumor-bearing mice treated with TET. a MDATO/OSM tumor-bearing mice treated with tetracycline (+TET) lost, on average, 11.4% of their body weight during TET treatment, compared with −TET mice, which gained an average of 5.5% of their body weight over the same period. b Representative image of mice with MDATO/OSM tumors +TET shows prominent spinal column, muscle wasting, and lack of visible adipose tissue. c Gross morphology of normal (left) and abnormal kidneys (right). Normal kidneys have a distinct border between the medulla and the cortex, with the cortex shown in a darker pink/red color and the medulla shown in a lighter pink color. This indicates that normal blood perfusion was taking place. Abnormal kidneys were either both pale and hypoperfused (middle) or damaged (right), with no clear distinction between the cortex and the medulla. d One hundred percent of mice in the +TET group have abnormal kidney morphologies, whereas only 25% of the mice in the −TET group have abnormal kidneys. **p < 0.01 by Fisher’s exact test. e Sera from mice with abnormal kidneys have a statistically significant higher level of OSM than sera from mice with normal kidneys. Data are expressed as mean ± SEM. *p < 0.05, **p < 0.01, and ***p < 0.001 by two-tailed Student’s t test. (ZIP 135 kb) [file 13058_2018_971_MOESM4_ESM.pptx]

## Slide 1
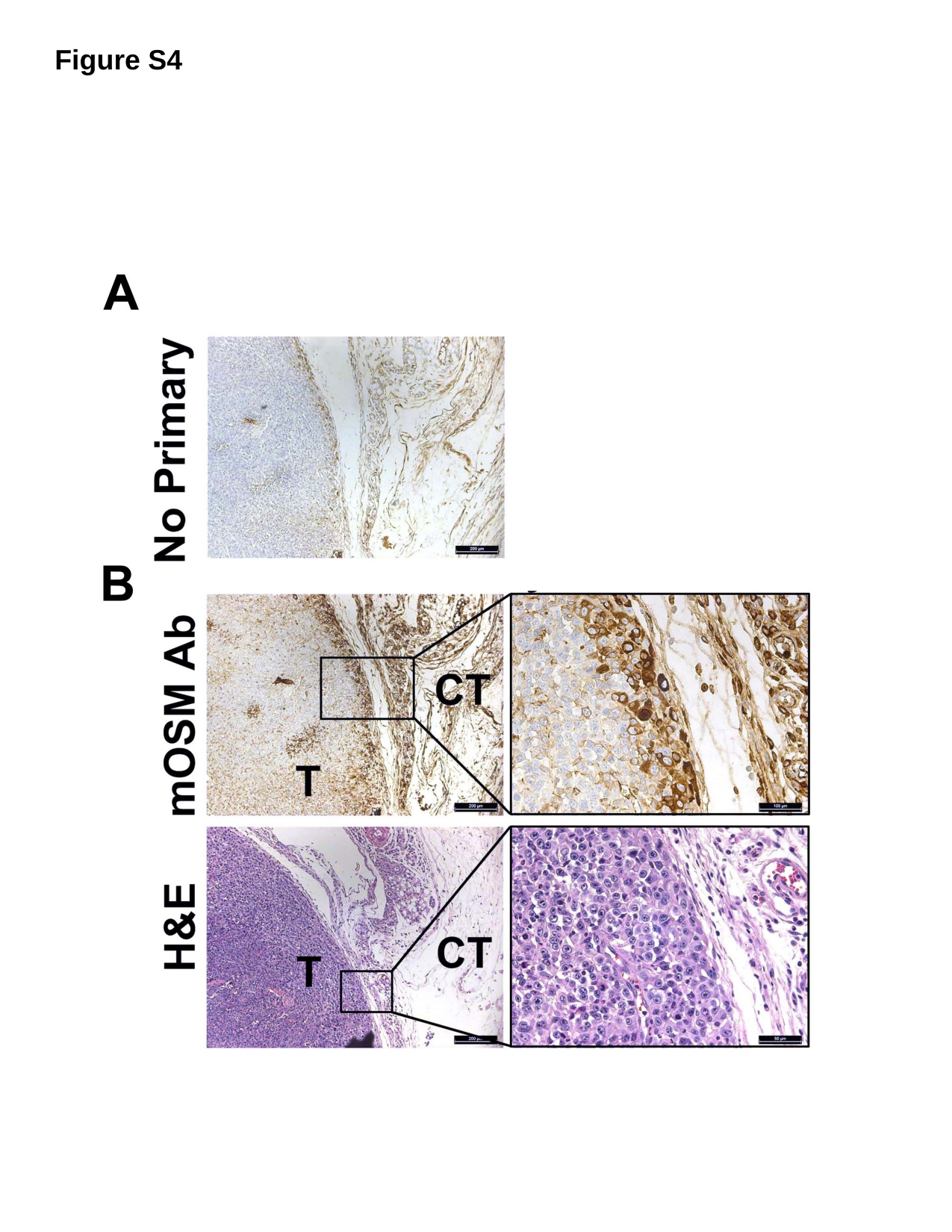

Figure S4

Supplement: Supplementary file 5 — Figure S4. OSM is highly expressed in orthotopic 4T1.2 primary mammary tumors in female BALB/c mice. Histology using H&E confirmed the presence of a large primary mammary tumor (T) 32 days after 4T1.2 mouse mammary tumor cell injection into the fourth mammary fat pad of female BALB/c mice. High OSM expression is seen in the tumor, as is background expression in the normal breast connective tissue (CT). OSM expression is shown to be highest in the invasive edge of the tumor (T) closest to the normal breast connective tissue (CT). Control slides with no primary OSM antibody show low background staining. (PPTX 315 kb) [file 13058_2018_971_MOESM5_ESM.pptx]

## Slide 1
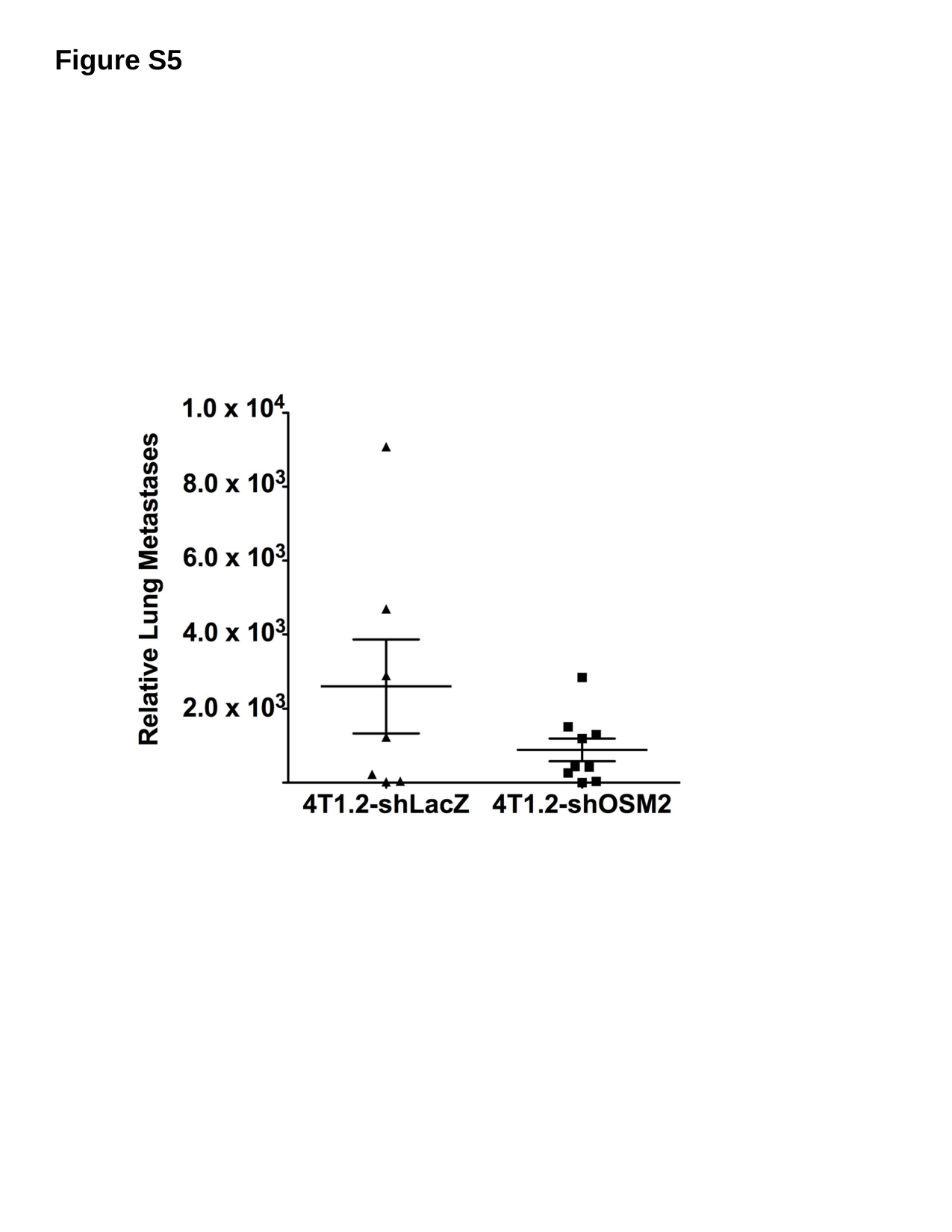

Figure S5

Supplement: Supplementary file 6 — Figure S5. qPCR analysis of lung metastases after intracardiac injections. 4T1.2-shLacZ cells and 4T1.2-shOSM2 cells were introduced via intracardiac injection, and qPCR analysis of the lung metastases indicated that the difference between the groups was not significant by two-tailed Student’s t test. (ZIP 60 kb) [file 13058_2018_971_MOESM6_ESM.pptx]

## Slide 1
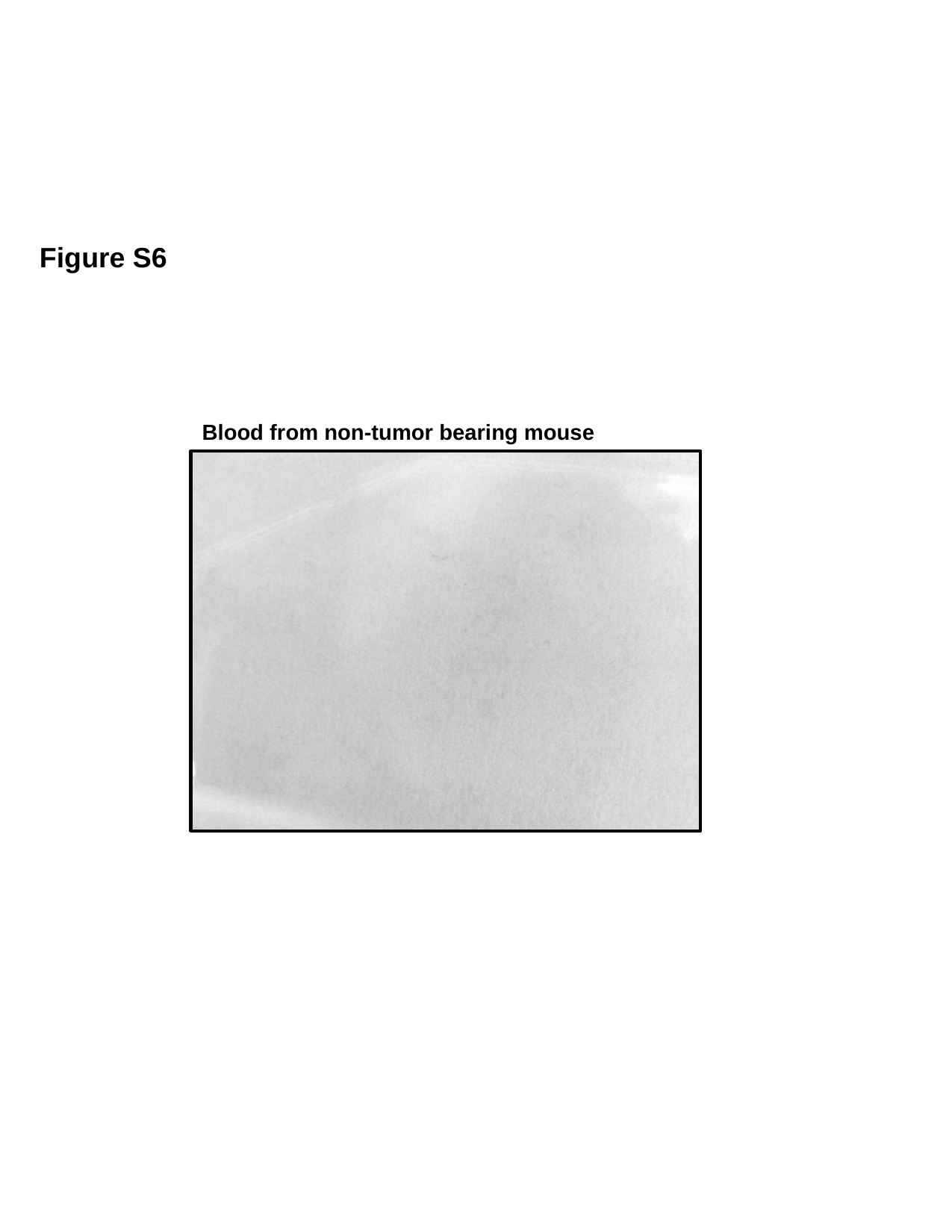

Figure S6
Blood from non-tumor bearing mouse

Supplement: Supplementary file 7 — Figure S6. Control colony-forming assay results derived from non-tumor-bearing mice. Blood from non-tumor-bearing mice contained no cells that formed colonies. (PPTX 53 kb) [file 13058_2018_971_MOESM7_ESM.pptx]

## Slide 1
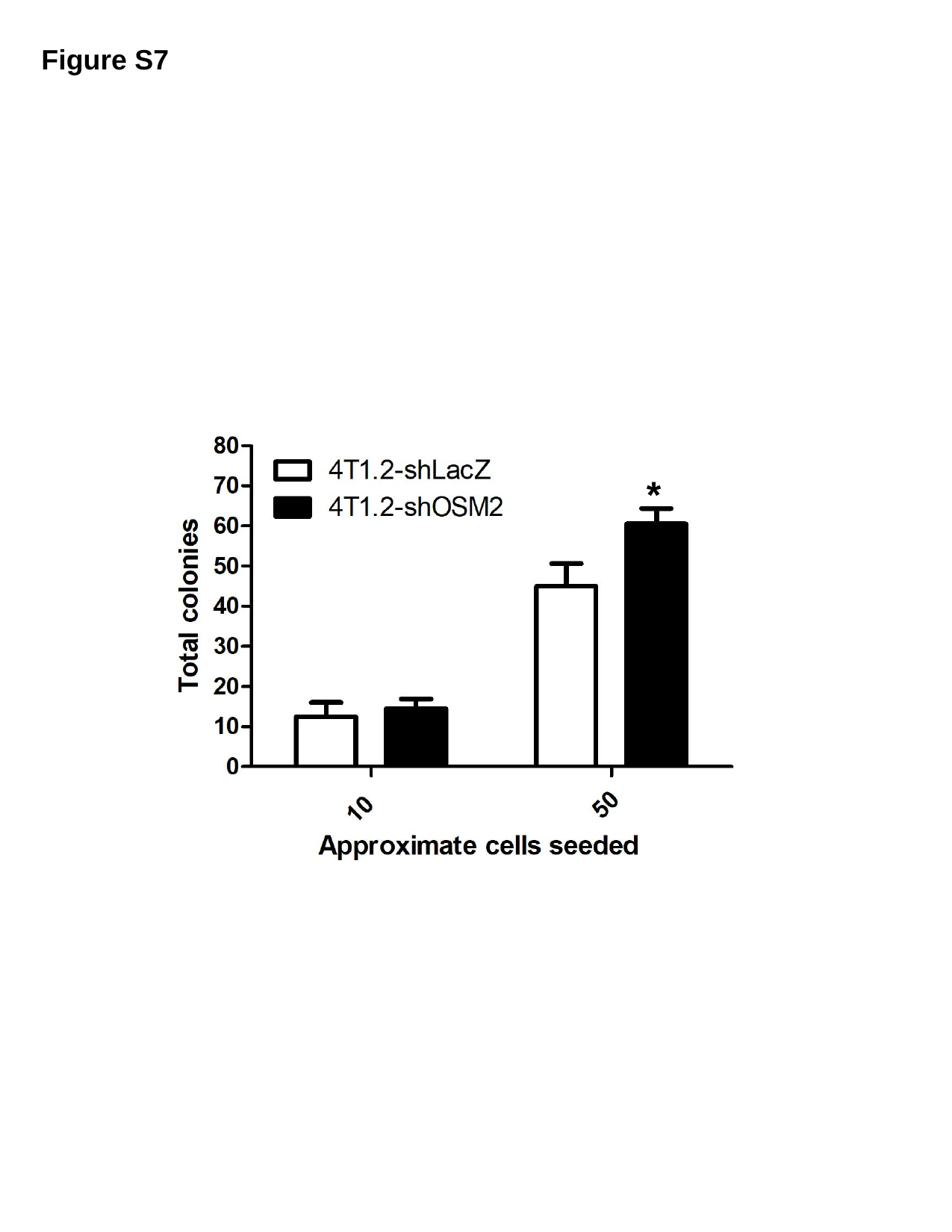

Figure S7
*

Supplement: Supplementary file 8 — Figure S7. Test of cell line-specific variance in colony-forming assay between 4T1.2-shLacZ and 4T1.2-shOSM2 cell lines. Approximately 10 and 50 cells of 4T1.2-shLacZ or 4T1.2-shOSM2 cells were seeded onto tissue culture plates and were allowed to incubate until colony formation. No significant differences between the cells were detected with ~ 10 cells seeded; however, there was a small but significant increase in the number of colonies with 4T1.2-shOSM2 cells at 50 cells seeded. Data are expressed as mean ± SEM. *p < 0.05 by one-way ANOVA with Bonferroni’s multiple comparisons test. (PPTX 68 kb) [file 13058_2018_971_MOESM8_ESM.pptx]

## Slide 1
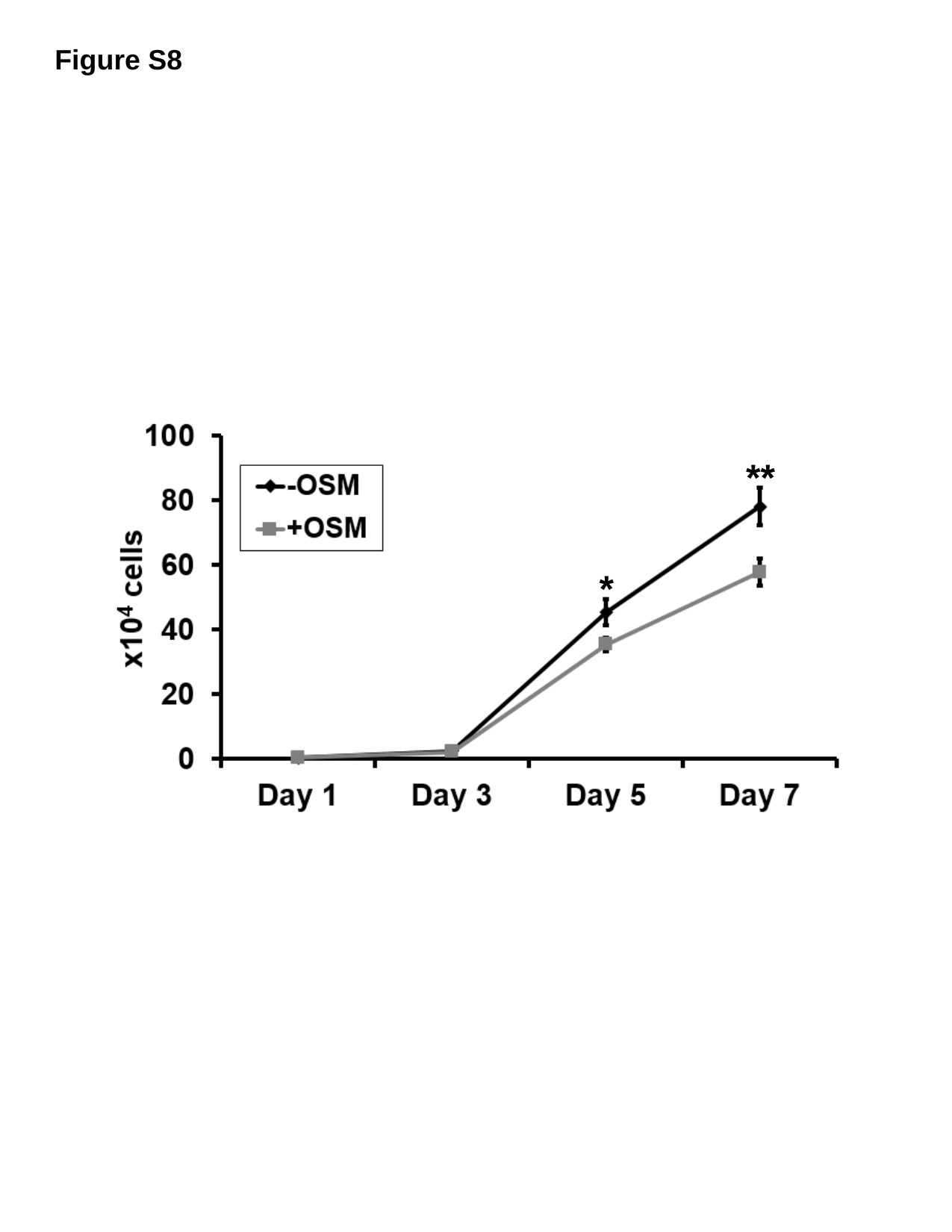

Figure S8
**
*

Supplement: Supplementary file 9 — Figure S8. OSM inhibits proliferation of 4T1.2 cells. One hundred 4T1.2 cells were plated at day 0 and treated with 25 ng/ml of OSM. By day 7, there was a 20% reduction in total cell numbers in the OSM-treated group versus the non-OSM-treated group. Data are expressed as mean ± SEM. *p < 0.05, **p < 0.01; statistical analysis was performed for each day using a two-tailed Student’s t test. (PPTX 21 kb) [file 13058_2018_971_MOESM9_ESM.pptx]
